# Costs of Tuberculosis at 3 Treatment Centers, Canada, 2010–2016

**DOI:** 10.3201/eid2809.220092

**Published:** 2022-09

**Authors:** Jonathon R. Campbell, Placide Nsengiyumva, Leslie Y. Chiang, Frances Jamieson, Hadeel Khadawardi, Henry K.-H. Mah, Olivia Oxlade, Hayden Rasberry, Elizabeth Rea, Kamila Romanowski, Natasha F. Sabur, Beate Sander, Aashna Uppal, James C. Johnston, Kevin Schwartzman, Sarah K. Brode

**Affiliations:** McGill University, Montreal, Quebec, Canada (J.R. Campbell, P. Nsengiyumva, O. Oxlade, A. Uppal, K. Schwartzman);; Research Institute of the McGill University Health Centre, Montreal (J.R. Campbell, P. Nsengiyumva, H. Rasberry, A. Uppal, K. Schwartzman);; McGill International TB Centre, Montreal (J.R. Campbell, O. Oxlade, K. Schwartzman);; British Columbia Centre for Disease Control, Vancouver, British Columbia, Canada (L.Y. Chiang, K. Romanowski, J.C. Johnston);; University of Toronto, Toronto, Ontario, Canada (F. Jamieson, E. Rea, B. Sander, S.K. Brode);; Public Health Ontario, Toronto (F. Jamieson, B. Sander);; West Park Healthcare Centre, Toronto (H. Khadawardi, H.K.-H. Mah, N.F. Sabur, S.K. Brode);; Toronto Public Health, Toronto (E. Rea);; University of British Columbia, Vancouver (K. Romanowski, J.C. Johnston);; St. Michael’s Hospital, Toronto (N.F. Sabur);; University Health Network, Toronto (B. Sander, S.K. Brode);; Montreal Chest Institute, Montreal (K. Schwartzman)

**Keywords:** tuberculosis and other mycobacteria, cost assessment, antimicrobial resistance, respiratory infections, bacteria, bacterial infections, Canada

## Abstract

We estimated costs of managing different forms of tuberculosis (TB) across Canada by conducting a retrospective chart review and cost assessment of patients treated for TB infection, drug-susceptible TB (DS TB), isoniazid-resistant TB, or multidrug-resistant TB (MDR TB) at 3 treatment centers. We included 90 patients each with TB infection and DS TB, 71 with isoniazid-resistant TB, and 62 with MDR TB. Median per-patient costs for TB infection (in 2020 Canadian dollars) were $804 (interquartile range [IQR] $587–$1,205), for DS TB $12,148 (IQR $4,388–$24,842), for isoniazid-resistant TB $19,319 (IQR $7,117–$41,318), and for MDR TB $119,014 (IQR $80,642–$164,015). Compared with costs for managing DS TB, costs were 11.1 (95% CI 9.1–14.3) times lower for TB infection, 1.7 (95% CI 1.3–2.1) times higher for isoniazid-resistant TB, and 8.1 (95% CI 6.1–10.6) times higher for MDR TB. Broadened TB infection treatment could avert high costs associated with managing TB disease.

After marked declines in tuberculosis (TB) incidence in Canada during the second half of the 20th century (*1*), progress toward elimination has stalled (*2*). Although a focus on detection and treatment of TB disease was highly effective in the past, changing epidemiology has limited the impact of this approach in reaching elimination. Additional approaches are needed. These approaches may include more targeted efforts for disproportionately affected populations, such as some Indigenous communities (*2*,*3*) and persons born outside of Canada (*4*).

Yet health resources are scarce (*5*). A fundamental aspect of decision-making in health is understanding the trade-offs associated with potential interventions or programs in comparison to other interventions and programs within the broader health agenda. To achieve the greatest return (improved health) on investment (money spent), policymakers should have accurate cost estimates for the various elements of TB prevention and care. However, costs associated with TB in Canada have not been estimated since 2004 (*6*). With new tests and treatments available for TB infection and disease, updated cost estimates will support informed decision-making for resource allocation around existing and emerging interventions and programs (*7*–*13*).

We sought to estimate the TB-related health system costs associated with managing persons treated for TB infection and different forms of TB disease, and the predictors of these costs, at 3 major TB treatment centers in British Columbia, Ontario, and Quebec, Canada.

## Methods

### Study Design and Participating TB Treatment Centers

We conducted a retrospective chart review of persons initiating treatment for TB infection, drug-susceptible TB (DS TB) disease, isoniazid-resistant TB disease, or multidrug-resistant TB (MDR TB) disease; we defined MDR TB as TB resistant to at least isoniazid and rifampin. We extracted data at 3 TB treatment centers in Canada: the British Columbia Centre for Disease Control (BCCDC), West Park Healthcare Centre (WPHC) in Toronto, Ontario, and the Montreal Chest Institute (MCI) in Quebec. In Canada, healthcare, including TB management, is a provincial and territorial responsibility.

BCCDC operates 2 TB clinics in the greater Vancouver region, treating all persons with TB infection and TB disease in the region. In 2016, BCCDC treated 241 persons for TB disease (all forms) and 676 persons for TB infection. WPHC, a rehabilitation and complex care hospital in Toronto, Ontario, housing a 20-bed dedicated inpatient TB unit and an ambulatory TB clinic, is recognized as a referral center for complex and drug-resistant TB. WPHC treated 119 persons for TB disease (all forms) and 33 persons for TB infection in 2016. MCI is located within the McGill University Health Centre, and is a center for TB screening and surveillance for newly arrived adult migrants to Canada. MCI treated 51 persons for TB disease (all forms) and 488 persons for TB infection in 2016.

### Study Inclusion and Exclusion Criteria

We included persons of any age who initiated treatment at any participating site during July 1, 2010–June 30, 2016; we reviewed consecutive patients, working backward from the end date, to permit adequate time to complete treatment and follow-up owing to the approximate 18–20-month duration of MDR TB treatment. All forms of TB disease required microbiologic confirmation (i.e., positive culture or positive nucleic acid amplification test). In addition, DS TB required confirmed susceptibility by phenotypic or genotypic means to all first-line TB drugs (i.e., isoniazid, rifampin, ethambutol, and pyrazinamide); isoniazid-resistant TB required confirmed resistance to isoniazid and susceptibility to rifampin; and MDR TB required confirmed resistance to at least isoniazid and rifampin. We excluded persons who initiated treatment at a participating site but later transferred to another treatment site where we could not access their charts.

For MDR TB disease, all persons meeting inclusion criteria at each site were included because of the low incidence in Canada. For TB infection, DS TB disease, and isoniazid-resistant TB disease, incidence is higher and treatment is more standardized; at each site we included up to 30 consecutive persons meeting inclusion criteria (*14*). This group included patients who had initiated treatment closest to June 30, 2016, for WPHC and MCI, and closest to December 31, 2015, for BCCDC.

### Procedures

For each person, we entered data into standardized forms (Appendix Table 1). In brief, for each person we collected detailed information on demographic and clinical characteristics, TB-related diagnostic tests performed, TB-related monitoring tests performed, TB-related inpatient and outpatient visits (including any visits requiring specialists), TB medication dose, frequency, and duration, including adverse events (and, if applicable, reasons for discontinuation), method of treatment administration (directly observed vs. self-administered), adjunct medications administered during treatment, use of interpreters, number of contacts traced (for all groups except those with TB infection), and posttreatment monitoring visits and evaluations. We completed data extraction during August 2018–May 2020.

At each site, we tabulated costs for services, consumables, and overheads (Appendix Table 2). We documented costs from the health system perspective in 2020 Canadian dollars (1.00 CAD ≈ 0.75 USD). When a cost item was unavailable from a given center, we used the mean from the other centers to impute it (Appendix Table 2). To determine drivers of cost, we grouped costs in 5 different categories: diagnosis, treatment, posttreatment follow-up, hospitalization, and public health costs. We did not include costs associated with healthcare seeking before TB diagnosis or for post-TB disease complications. To estimate true resource use, we performed microcosting where possible; in all other cases, we used top-down approaches.

In the diagnosis category, we performed microcosting and considered costs associated with initial physician consultations, nurse and interpreter time, and overheads, as well as costs of diagnostic tests (e.g., tuberculin skin test, chest radiograph, smear microscopy, sputum culture, drug-susceptibility testing, and computed tomography scans) and of routine screening for other related conditions (e.g., HIV infection and viral hepatitis).

In the treatment category, we performed microcosting and considered costs associated with TB and adjunct medications, tests for treatment and adverse event monitoring (e.g., for liver transaminases, complete blood count, therapeutic drug monitoring, and audiometry), tests for treatment response (e.g., sputum culture), and personnel and overhead associated with follow-up visits with nurses, physicians, and specialists. Bedaquiline and clofazimine are given under compassionate-use programs in Canada and are not associated with costs to programs.

In the posttreatment follow-up category, we performed microcosting and only considered costs associated with surveillance for TB recurrence. These costs included chest imaging and costs of routine follow-up appointments.

In the hospitalization category, we performed microcosting and considered per-diem costs attributed to each day of hospitalization according to setting. We also considered costs associated with visits by physicians during the stay and with investigations and medications.

In the public health category, we considered costs of delivering directly observed therapy (DOT), when performed, and costs associated with contact investigation. For costs of delivering DOT, we performed microcosting at MCI and BCCDC, considering personnel (nurse, pharmacist, or both) and other costs (e.g., travel). We used a top-down approach at WPHC on the basis of data from Toronto Public Health. Because of the varied nature of contact investigations across sites, we used a top-down approach on the basis of data from Toronto Public Health because they had the most systematic and comprehensive data for contact investigation (Appendix).

### Data Analysis

We performed descriptive analysis of patient characteristics by TB treatment center and form of TB (TB infection, DS TB, isoniazid-resistant TB, or MDR TB). For persons with TB infection, we also described those receiving different regimens: 9 months of isoniazid, 4 months of rifampin, or other isoniazid-containing regimens. For persons with MDR TB, we further described persons with additional resistance to a fluoroquinolone (ofloxacin, moxifloxacin, or levofloxacin), resistance to a second-line injectable drug (amikacin, kanamycin, or capreomycin), or both.

For each person, we used the itemized costs to estimate the costs associated with each cost category defined previously and summed them to arrive at an overall cost. We estimated median costs and interquartile range (IQR) to illustrate cost variation, but also estimated mean costs, because these data are most useful for policymakers. We estimated costs for each form of TB overall and in different subgroups (as relevant): sex, age at treatment initiation (dichotomous, based on median age in all persons), presence versus absence of adverse events causing drug cessation, duration of hospitalization (dichotomous, based on median hospitalization duration in all persons hospitalized), completion of treatment, acid-fast bacilli smear status, presence of cavities, and location of TB disease.

We performed regression by using linear mixed models to identify predictors of cost for all forms of TB together (using DS TB as the reference category). We conducted a subgroup analysis where we excluded TB infection to assess the impact of clinical characteristics such as radiography and microbiologic findings. We also conducted stratified analyses for each form of TB separately. We treated each site as a random intercept. For each analysis, we log-transformed costs and performed univariable analysis on several predictors (Appendix Table 3). We included age and sex as a priori predictors in all multivariable models and any predictor with a p value <0.2 in univariable analysis. We back-transformed the resultant estimates and 95% CIs, which we interpreted as cost ratios (*15*). Because costs are probably associated with treatment completion or noncompletion, we did a post hoc sensitivity analysis, in which we repeated all analyses but excluded persons who did not complete treatment. We performed all analyses in R version 4.1.0 (*16*) using package lme4 (version 1.1–23) (*17*).

This study was approved by the research ethics boards of the sites where data were collected. These boards were the Research Institute of the McGill University Health Centre (approval no. 2019-4811), the University of British Columbia (approval no. H18-01700), and West Park Healthcare Centre (approval no. 18-017-WP).

## Results

### Total Population

We included a total of 313 persons in the study: 101 (32%) from BCCDC, 132 (42%) from WPHC, and 80 (26%) from MCI. We tabulated the characteristics of included persons (Table 1) and the estimated costs of their management, stratified by form of TB (Table 2). We also stratified costs by patient characteristics (Appendix Tables 4–7). We determined mean costs for all analyses (Appendix Table 8). Overall, the median cost of TB infection was $804 (IQR $587–$1,205), of DS TB disease was $12,148 (IQR $4,388–$24,842), of isoniazid-resistant TB disease was $19,319 (IQR $7,117–$41,318), and of MDR TB disease was $119,014 (IQR $80,642–$164,015).

**Table 1:** Characteristics of patients initiating treatment for different forms of TB at 3 treatment centers, Canada, July 2010–June 2016*

Characteristic	No. (%)			
	TB infection, n = 90	DS TB, n = 90	INHR TB, n = 71	MDR TB, n = 62
TB treatment center, province				
British Columbia Centre for Disease Control	30 (33)	30 (33)	30 (42)	11 (18)
West Park Healthcare Centre, Ontario	30 (33)	30 (33)	27 (38)	45 (73)
Montreal Chest Institute, Quebec	30 (33)	30 (33)	14 (20)	6 (9)
Year of treatment initiation				
2010–2011	0 (0)	0 (0)	12 (17)	10 (16)
2012–2013	1 (1)	0 (0)	13 (18)	19 (31)
2014	15 (17)	1 (1)	20 (28)	15 (24)
2015	42 (47)	57 (64)	22 (31)	13 (21)
2016	32 (35)	32 (35)	4 (6)	5 (8)
Age				
Median (IQR) age, y	36 (31–49)	43 (30–62)	44 (31–61)	32 (27–47)
Sex				
F	55 (61)	50 (56)	38 (54)	34 (55)
M	35 (39)	40 (44)	33 (46)	28 (45)
Nativity				
Born in Canada	11 (12)	10 (11)	9 (13)	5 (8)
Born outside Canada	79 (88)	80 (89)	62 (87)	57 (92)
HIV status				
Positive	0	1 (1)	0 (0)	1 (2)
Negative	33 (37)	69 (77)	12 (17)	59 (95)
Unknown	57 (63)	20 (22)	59 (83)	2 (3)
Diabetes				
Has diabetes	12 (13)	13 (14)	10 (14)	10 (16)
Does not have diabetes	74 (82)	75 (83)	60 (85)	52 (84)
Unknown	4 (4)	2 (2)	1 (1)	0
Hospitalization Information				
Hospitalized	0	46 (51)	47 (66)	60 (97)
Median (IQR) duration, d	NA	24 (9–36)	23 (17–69)	99 (66–159)
Treatment information				
Median (IQR) duration, mo	5.8 (4.0–9.0)	8.9 (6.1–9.6)	11.7 (9.1–16.7)	21.2 (20.0–24.7)
Had to stop >1 drug because of adverse event	7 (8)	38 (42)	29 (41)	52 (84)
Median (IQR) drugs stopped because of adverse event	0 (0–0)	0 (0–1)	0 (0–1)	2 (1–3)
Cure or treatment complete	77 (86)	83 (92)	63 (89)	49 (79)
Incomplete treatment	13 (14)	7 (8)	8 (11)	13 (21)
Clinical characteristics				
Pulmonary TB only	NA	68 (76)	51 (72)	47 (76)
Extrapulmonary TB	NA	22 (24)	20 (28)	15 (24)
AFB smear positive	NA	47 (52)	35 (49)	22 (35)
Cavities on chest x-ray	NA	30 (33)	21 (30)	15 (24)
Public health characteristics				
Used directly observed therapy	NA	32 (36)	33 (46)	54 (86)
Mean (range) no. contacts	NA	4 (0–30)	8 (0–224)	4 (0–97)

*Data are no. (%) except as indicated. AFB, acid-fast bacilli; DS, drug-susceptible; INHR, isoniazid-resistant; IQR, interquartile range; MDR, multidrug-resistant; NA, not applicable; TB, tuberculosis.

**Table 2:** Total costs and component costs of managing different forms of TB at 3 treatment centers, Canada, July 2010–June 2016*

Characteristic	Cost, in 2020 Canadian dollars			
	TB infection, n = 90	DS TB, n = 90	INHR TB, n = 71	MDR TB, n = 62
Median (IQR) costs†				
Total costs	804 (587–1,205)	12,148 (4,388–24,842)	19,319 (7,117–41,318)	119,014 (80,642–164,015)
Diagnosis	267 (217–376)	701 (526–1,026)	819 (657–1,049)	1,083 (925–1,331)
Treatment	521 (377–771)	2,145 (1,614–3,187)	2,864 (2,263–3,919)	61,426 (29,840–108,703)
Posttreatment monitoring	0 (0–0)	139 (28–283)	130 (39–195)	193 (39–341)
Hospitalization	0 (0–0)	2,600 (0–15,524)	10,400 (0–27,227)	41,216 (35,178–55,766)
Associated with public health interventions	0 (0–0)	3,174 (632–5,232)	2,885 (1,111–6,174)	6,399 (4,657–6,798)
Mean costs‡				
Total costs	917	15,772	32,343	131,780
Diagnosis	308	789	860	1,233
Treatment	587	2,585	4,641	74,709
Posttreatment monitoring	22	181	166	243
Hospitalization	0	8,587	19,963	48,791
Associated with public health interventions	0	3,630	6,713	6,804

*AFB, acid-fast bacilli; DS, drug-susceptible; INHR, isoniazid-resistant; IQR, interquartile range; MDR, multidrug-resistant; TB, tuberculosis. †Component values may not sum to the total cost value because of use of medians. ‡Component values may not sum to the total cost value because of rounding.

We determined the relative contribution of each cost category to the overall cost of management, again stratified by form of TB (Figure). Although diagnosis costs were a substantial contributor to overall costs in TB infection, their contribution was comparatively smaller for other forms of TB. For TB disease (DS TB, isoniazid-resistant TB, and MDR TB), hospitalization costs accounted for a substantial proportion of all costs (54.4% for DS TB, 61.7% for isoniazid-resistant TB, and 37.2% for MDR TB).

**Figure:**
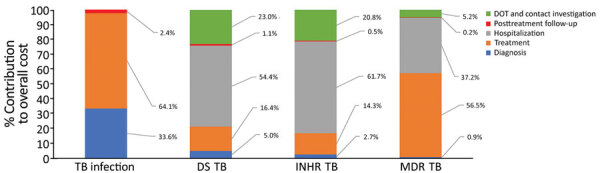
Relative contribution of each cost category to overall cost of managing different forms of TB at 3 treatment centers, Canada, July 2010–June 2016. DOT, directly observed therapy; DS, drug-susceptible; INHR, isoniazid-resistant; MDR, multidrug-resistant; TB, tuberculosis.

Among the 313 persons, multivariable regression estimated costs of managing TB infection were 11.1 times lower (adjusted cost ratio 0.09 [95% CI 0.07–0.11]) than costs of managing DS TB. Conversely, costs of managing isoniazid-resistant TB were 1.7 times higher (95% CI 1.3–2.1) than DS TB, whereas costs of managing MDR TB were 8.1 times higher (95% CI 6.1–10.6) than DS TB (Table 3; univariable regression results [Appendix Table 9]). When we excluded TB infection from multivariable regression and included clinical characteristics (Appendix Table 10), adjusted cost ratios were reduced for isoniazid-resistant TB (1.3 [95% CI 1.1–1.7]) and MDR TB (3.6 [95% CI 2.6–5.1]). Estimates were not substantially different when we excluded persons who did not complete treatment (Appendix Table 11).

**Table 3:** Multivariable analysis of characteristics associated with increasing or decreasing costs of managing different forms of TB at 3 treatment centers, Canada, July 2010–June 2016*

Characteristic	Cost ratio (95% CI)				
	All patients, n = 313	TB infection, n = 90	DS TB, n = 90	INHR TB, n = 71	MDR TB, n = 62
TB type					
DS TB	Referent	NA	NA	NA	NA
TB infection	0.09 (0.07–0.11)	NA	NA	NA	NA
INHR TB	1.7 (1.3–2.1)	NA	NA	NA	NA
MDR TB	8.1 (6.1–10.6)	NA	NA	NA	NA
Age group, y					
<40	Referent	Referent	Referent	Referent	Referent
>40	0.97 (0.8–1.2)	1.3 (1.1–1.5)	0.9 (0.6–1.3)	1.2 (0.9–1.6)	0.9 (0.7–1.1)
Sex					
F	Referent	Referent	Referent	Referent	Referent
M	0.9 (0.8–1.1)	0.99 (0.8–1.2)	0.8 (0.6–1.1)	1.01 (0.8–1.4)	0.98 (0.8–1.3)
HIV					
HIV-negative or unknown	Referent	NA†	Referent	NA†	NA‡
HIV-positive	1.8 (0.6–5.4)	NA†	11.9 (2.7–52.0)	NA†	NA‡
Diabetes					
No diabetes or unknown	NA‡	NA‡	NA‡	Referent	NA‡
Has diabetes	NA‡	NA‡	NA‡	1.4 (0.9–2.2)	NA‡
Adverse events causing drug stop					
None	Referent	NA‡	Referent	Referent	NA‡
Because of >1 drug	1.4 (1.1–1.7)	NA‡	1.5 (1.03–2.0)	1.2 (0.9–1.7)	NA‡
Hospitalization					
None or <2 mo	NA	NA	Referent	Referent	Referent
>2 mo	NA	NA	3.7 (1.9–7.4)	3.2 (2.1–4.7)	1.5 (1.1–2.0)
AFB smear					
Negative or unknown	NA	NA	Referent	Referent	Referent
Positive	NA	NA	1.5 (0.98–2.2)	1.3 (0.9–1.7)	1.02 (0.8–1.3)
Cavities on chest radiograph					
No or unknown	NA	NA	Referent	NA‡	Referent
Yes	NA	NA	1.2 (0.8–1.8)	NA‡	1.3 (0.96–1.8)
TB location					
Pulmonary only	NA	NA	Referent	NA‡	NA‡
Extrapulmonary involvement	NA	NA	0.7 (0.5–1.1)	NA‡	NA‡
No. contacts					
Per additional contact	NA	NA	1.05 (1.02–1.08)	1.02 (1.01–1.02)	1.01 (0.99–1.01)
Received DOT					
No	NA	NA	NA‡	Referent	Referent
Yes	NA	NA	NA‡	2.0 (1.2–3.3)	0.8 (0.5–1.3)
TB infection regimen					
Mono-rifampin	NA	Referent	NA	NA	NA
Isoniazid-containing	NA	1.3 (0.97–1.7)	NA	NA	NA
MDR TB resistance pattern					
MDR TB	NA	NA	NA	NA	Referent
Fluoroquinolone-resistance, SLI resistance, or both	NA	NA	NA	NA	1.4 (1.02–2.0)

*AFB, acid-fast bacilli; DOT, directly observed therapy; DS, drug-susceptible; INHR, isoniazid-resistant; IQR, interquartile range; MDR, multidrug-resistant; NA, not applicable; SLI, second-line injectable; TB, tuberculosis. †No persons living with HIV in patient group. ‡Not retained in multivariable model because p>0.2 in univariable analysis.

### TB Infection

Overall, we included 90 persons treated for TB infection (30 at each center) (Table 1). Of these persons, 53 (59%) initiated 9 months of isoniazid, 35 (39%) initiated 4 months of rifampin, and 2 (2%) initiated isoniazid and rifampin (Appendix Tables 12, 13).

Approximately two-thirds of costs for TB infection were associated with treatment (Figure); absolute treatment costs were correlated with duration (Appendix Table 13). Persons initiating an isoniazid-containing regimen had overall costs 1.3-times (95% CI 0.98–1.7) higher than persons initiating a rifampin-only regimen (Table 3).

### DS TB Disease

We included 90 persons treated for DS TB disease (30 at each center) (Table 1). Approximately half (46 [51%]) were hospitalized for a median duration of 24 (IQR 9–36) days. The median duration of treatment was 8.9 (IQR 6.1–9.6) months; treatment was shorter for persons who were smear-negative and without cavities (6.6 months [IQR 6.1–9.1]) compared with persons who were smear-positive or had cavities, or both (9.1 months [IQR 6.4–10.0]) (Appendix Table 14).

More than half the cost of DS TB disease management was related to hospitalization, whereas approximately one third reflected contact investigations and DOT administration (Figure). Costs of managing DS TB disease were much lower at MCI (median $4,987) than at WPHC ($13,328) and BCCDC ($15,201), largely because of variation in disease severity and attendant differences in hospitalization among persons treated at these centers (Appendix Table 14). Costs were 3.7 (95% CI 1.9–7.4) times higher for persons hospitalized for >2 months compared with persons not hospitalized at all or hospitalized <2 months (Table 3).

### Isoniazid-Resistant TB Disease

We included 71 persons treated for isoniazid-resistant TB disease (30 at BCCDC, 27 at WPHC, and 14 at MCI) (Table 1). Of those, 47 (66%) were hospitalized, with median duration 23 (IQR 17–69) days. The median treatment duration was 11.7 (IQR 9.1–16.7) months and varied substantially by TB treatment center (Appendix Table 15). Fifty-four (76%) persons received regimens containing a fluoroquinolone, and 8 (11%) received a second-line injectable (Appendix Table 15).

Over 60% of costs associated with isoniazid-resistant TB disease were because of hospitalization (Figure). Treatment was shortest and costs lowest at MCI (median duration 8 months; median cost $6,504) and treatment longest and costs highest at WPHC (median duration 17.6 months; median cost $34,400). Costs were 3.2 (95% CI 2.1–4.7) times higher for persons hospitalized >2 months compared with patients not hospitalized at all or hospitalized <2 months (Table 3).

### MDR TB Disease

We included 62 persons treated for MDR TB disease (11 at BCCDC, 45 at WPHC, and 6 at MCI) (Table 1). Of these, 2 (3%) had additional fluoroquinolone resistance, 6 (10%) had additional resistance to a second-line injectable, and 4 (6%) had both. Nearly all (60 [97%]) were hospitalized for a median duration of 99 (IQR 66–159) days. The median treatment duration was 21.2 (IQR 20.0–24.7) months and was similar across centers (Appendix Table 16). About half (34 [55%]) of the patients received linezolid, whereas few received the newer drugs bedaquiline (3 [5%]) or delamanid (4 [6%]) (Appendix Tables 16, 17).

Costs associated with treatment (56.5%) and hospitalization (37.2%) were the largest cost components for MDR TB management (Figure). In adjusted analyses, resistance to a fluoroquinolone, a second-line injectable, or both were associated with 1.4 (95% CI 1.02–2.0) times higher costs (Table 3).

We analyzed median duration and cost of each medication received (Table 4). Cycloserine was the most expensive drug, costing a median of $57,658 (IQR $28,942–$91,935) per person. New and repurposed drugs (i.e., linezolid, delamanid, and carbapenems) were also expensive (median cost range $8,459–$22,437). Fluoroquinolones and second-line injectables were less expensive (median cost range $330–$4,024). Compassionate-use drugs (clofazimine and bedaquiline) did not contribute to costs to TB programs.

**Table 4:** Duration and costs of drugs used among patients initiating treatment for MDR-TB disease (n = 62) at 3 treatment centers, Canada, July 2010–June 2016*

Drug	No. (%) patients receiving drug	Median (IQR) duration, mo	Cost, 2020 Canadian dollars	
			Median (IQR) cost per person	Mean cost per person
Amikacin	58 (94)	5.1 (2.6–8.2)	4,024 (2,629–8,479)	7,263
Moxifloxacin	58 (94)	19.9 (8–22.4)	809 (318–985)	698
Ethambutol	56 (90)	8.6 (1.1–15.9)	280 (51–637)	382
Pyrazinamide	53 (85)	3.1 (0.8–8.8)	217 (50–800)	545
Clofazimine	50 (81)	19.7 (6.8–22.6)	Given under compassionate use	
Isoniazid†	47 (76)	0.7 (0.4–1.7)	14 (9–35)	44
Para-amino salicylic acid	47 (76)	13.6 (3.2–20.4)	6,609 (1,917–11,411)	7,036
Rifampin†	46 (74)	0.7 (0.5–1.3)	15 (9–35)	37
Cycloserine	42 (68)	13.4 (7–20.6)	57,658 (28,942–91,935)	61,590
Ethionamide	40 (65)	11.3 (2.8–19.7)	691 (191–1,304)	785
Linezolid	34 (55)	8.4 (3.5–16.2)	10,057 (4,608–19,023)	12,070
Amoxicillin/clavulanate	20 (32)	14.3 (3.9–18.6)	1,144 (286–1,652)	1,524
Imipenem/cilastatin	14 (23)	6.1 (1.8–7.3)	8,459 (3,244–10,267)	7,855
Levofloxacin	13 (21)	11.4 (6.4–18.2)	330 (69–1,328)	1,022
Clarithromycin	10 (16)	16.8 (3.4–21.8)	2,711 (549–3,531)	2,226
Rifabutin	5 (8)	22.9 (22.7–23.7)	13,207 (11,490–13,341)	10,865
Delamanid	4 (6)	3.7 (1.6–6.9)	22,437 (5,616–38,475)	21,654
Azithromycin	3 (5)	9.5 (6.8–13.4)	41 (29–3,850)	2,572
Bedaquiline	3 (5)	5.5 (3.9–5.5)	Given under compassionate use	
Meropenem	3 (5)	2.1 (1.3–10.2)	21,123 (10,766–75,480)	50,456
Streptomycin	2 (3)	0.7 (0.4–1.1)	980 (512–1,448)	980

*IQR, interquartile range. †Stopped when resistance detected if treatment had been started before MDR TB confirmation.

## Discussion

At 3 TB treatment centers in Canada, we found costs of managing TB infection were modest compared with costs of managing TB disease. For persons with TB disease, duration of hospitalization and extent of drug resistance were major drivers of cost. Among the 3 TB treatment centers, treatment practices varied with respect to length of hospital stays and composition or duration of treatment regimens, perhaps because of variations in treatment philosophy, isolation practices, patient profiles, or a combination of these factors, which resulted in substantial cost differences between centers.

In 2004, the average health system cost of managing TB disease in Canada was estimated to be $25,986 per person (*6*,*18*). When applying our cost estimates against the distribution of drug-resistant TB disease in Canada (*2*,*19*), we estimate an average cost of $17,506. These differences appear to be influenced by variations in study aims and approaches. The 2004 study aimed to estimate all costs spent on TB services using a top-down approach, whereas our study aimed to estimate costs per patient initiated on treatment, largely by using microcosting approaches. For example, the 2004 study included costs associated with microbiologic testing of all persons tested for TB disease, not only those ultimately treated. In contrast, our study included costs associated with outpatient specialist consultations, additional tests, and adjunctive medications, which were not included in the 2004 study.

Direct costs associated with managing MDR TB disease in Canada appear to be substantially lower than estimates from the United States for 2005–2007 (*20*). When inflated and converted to 2020 Canadian dollars (*21*), direct costs associated with MDR TB disease are ≈$243,000, or 2.0-fold more expensive than comparative estimates from this study, whereas costs associated with MDR TB with additional resistance to a fluoroquinolone and second-line injectable are ≈$757,000, or 4.5-fold more expensive. These differences appear almost entirely driven by costs associated with hospitalization and inpatient care, as opposed to outpatient care.

This study highlights managing persons with evidence of TB infection is less costly than TB disease, particularly when using 4 months of rifampin (3 months of weekly isoniazid and rifapentine is not widely available in Canada). Hospitalization was a major driver of costs for TB disease; use of community care to prevent hospitalization may reduce overall costs (*22*). From our estimates, the total costs (including diagnosis, treatment, and posttreatment monitoring) of using 4 months of rifampin ($671 per person) for 23 persons with evidence of TB infection are equivalent to the total costs (including diagnosis, treatment, posttreatment monitoring, hospitalization, and public health interventions) of managing 1 person with DS TB disease ($15,771 per person). However, it is important to also consider costs associated with identifying persons who would benefit from TB preventive treatment in specific epidemiologic contexts, because these costs will affect the relative cost-effectiveness of preventive treatment.

Our study focused on persons initiating treatment for TB largely during 2015–2016, but new regimens have since become available. In 2018, the World Health Organization (WHO) recommended that persons with MDR TB disease treated with longer regimens should receive a fluoroquinolone, bedaquiline, linezolid, and >1 of clofazimine or cycloserine. Both clofazimine and bedaquiline are given under compassionate-use programs in Canada. However, a course of bedaquiline in Canada could cost $30,000 USD (*23*), whereas a course of clofazimine would cost approximately $600 USD (*24*,*25*). At these prices, the overall costs of treatment are unlikely to change, although regimens should be better tolerated (*26*). Shorter MDR TB regimens recommended by WHO (*27*) are not widely used in Canada. In 2021, the WHO conditionally recommended a moxifloxacin- and rifapentine-based 4-month regimen for DS TB disease (*28*). Despite a shorter treatment duration, costs are unlikely to be reduced in Canada because savings associated with reduced health visits and DOT will probably be outweighed by higher medication costs for rifapentine and moxifloxacin (*29*).

Our study’s first limitation is that costs were only considered from the health system perspective and for persons ultimately initiating treatment from the point when persons underwent diagnostic testing for TB. This approach excludes costs associated with prediagnosis healthcare seeking behavior, the long-term financial impacts associated with TB disease, and other patient costs such as lost income, travel, and childcare, which may be substantial (*30*–*32*). The TB treatment centers included in this study were prioritized so as to obtain robust estimates of the costs of treatment for drug-resistant TB disease; the 3 centers treated ≈60% of all MDR TB disease in Canada during the study period (*33*). Other forms of TB managed at the same centers allowed for instructive comparisons. We only could capture information contained in patient charts. Most notably absent were interactions with the health system before diagnosis, which may lead to an underestimation of costs. DOT for TB disease was rarely used at BCCDC and MCI. Costs associated with public health interventions are likely to be higher at centers performing routine, daily DOT. Although we conducted microcosting to estimate true resource use where possible, we had to use top-down approaches for some costs, which may overestimate true resource use. Last, not all costs were available at all centers, and imputed costs for some centers may not be precise, although cost imputation was rare.

A key strength of our study is the comprehensive nature of data collection with respect to healthcare utilization and associated costs, which permitted microcosting of many aspects of TB care and attendant insight into cost drivers and predictors. An additional strength is the separate estimation of costs for drug-resistant TB disease, including isoniazid-resistant and MDR TB, all managed in the same centers, filling a major data gap in Canada.

In summary, costs of managing TB disease increased substantially with drug resistance and were highest among persons hospitalized for >2 months; the costs of managing TB infection were comparatively much smaller. Because TB rates remain stagnant in Canada, these data will be useful for policymakers considering TB prevention and care interventions to support the overall goal of TB elimination.

Appendix Additional information about costs of tuberculosis at 3 treatment centers, Canada, 2010–2016.
