# Supplementary material for: Costs of Tuberculosis at 3 Treatment Centers, Canada, 2010–2016
Source: Emerg Infect Dis. 2022 Sep;28(9):1814–23. doi: 10.3201/eid2809.220092 (PMC9423918; doi:10.3201/eid2809.220092)
Supplement: Appendix — Additional information about costs of tuberculosis at 3 treatment centers, Canada, 2010–2016. [file 22-0092-Techapp-s1.pdf]

Article DOI: https://doi.org/10.3201/eid2809.220092

Costs of Tuberculosis at 3 Treatment Centers, Canada, 2010–2016

Appendix

Methods

Additional Details on Cost Estimation for Contact Investigations

Contact investigation costs were estimated using a top-down approach based on data from Toronto Public Health in 2017. Costs for contact investigations were estimated solely on time spent (i.e., no costs of overhead or materials) by public health staff who managed the 206 pulmonary tuberculosis cases in Toronto, Ontario in 2017. From these 206 cases, 1689 contacts were identified (i.e., 8.2 contacts per case). From interviews performed by Elizabeth Rea (who works at Toronto Public Health and is an author on this manuscript), public health nurse tuberculosis case managers estimated that they spent 40% of their time on activities related to contact investigation (follow-ups, counselling, testing, sputum collection, chest x-rays, and diagnosis). The total combined annual salary (including benefits) for the 22 FTE public health nurse case managers was $2,192,465, and thus, 40% of their time was valued at $876,986. In addition, there are support staff that estimated they spent 25% of their time assisting with data management, logistics, and communication associated with contact investigations. The total combined annual salary (including benefits) of the 5 FTE support staff was $390,104, and thus, 25% of their time was valued at $97,526. Taken together, this results in estimated personnel cost associated with contact investigation of $4,730 per index tuberculosis patient or a cost of $577 per contact identified. In terms of time spent on contact investigation by public health staff, if one assumes 20 working days per month (or 240 working days per year) then the time associated with contact investigation per index tuberculosis patient was 11.7 days or 1.43 days per contact identified.

Appendix Table 1. Data Fields for Patient Information

Variable	Value
Site ID	numeric
Patient ID	numeric
Patient Age (At Treatment Start)	numeric
Sex	Male, Female, Unknown
Foreign-Born	Yes, No
If Foreign-Born, What is the Country of Origin?	character
If Foreign-Born, What is the Year of Arrival?	numeric
If Foreign-Born, What is the Immigration Status?	Citizen, Permanent Resident, Temporary Permit, Refugee, Undocumented, Other
Occupation	Health care worker, Migrant or seasonal worker, Retired, Not seeking employment, Correctional facility employee, Military, Other, Unemployed, Undocumented, Student
If Employed, Had to Stop Working for Treatment	Yes, No, Unknown
Weight (kg)	numeric
BMI (in kg/m^2)	numeric
HIV Test Placed	Yes, No, Unknown
HIV Status	Positive, Negative, Unknown, Not Done
On ART	Yes, No, Unknown
Previous Treatment?	Yes, No, Unknown
Previous Treatment Type	TB Infection, DS-TB, MDR-TB, Unknown
Most Recent Previous Treatment Date	(mm/dd/yyyy)
Previous Treatment Outcome	Cure, Complete, Failure, Relapse, Lost, Adverse Event, Unknown
Previous Treatment with First-Line Drugs	Yes, No, Unknown
Previous Treatment with Second-Line Drugs	Yes, No, Unknown
Diabetes Status	Yes, No, Unknown
Smoking Status	Present, Ex, Never, Unknown
Alcohol Consumption	Yes, No, Unknown
Injectable Drug Use	Yes, No, Unknown
BCG Vaccinated	Yes, No, Unknown
Health Insurance	Yes, No, Unknown
Type of Health Insurance Coverage	Provincial, Interim Federal Health (Refugee Claimant), Private, None, Unknown
Patient Status at Diagnosis	Alive, Dead
Chest X-Ray Date	(mm/dd/yyyy)
Chest X-Ray Cavitation	Yes, No, Unknown, Not Done
Chest X-Ray Bilateral cavitation	Yes, No, Unknown, Not Done
Chest X-Ray Bilateral disease	Yes, No, Unknown, Not Done
Total Number of Chest X-Rays during diagnosis	numeric
Total Number of Chest X-Rays during treatment	numeric
Total Number of post-treatment Chest X-Rays	numeric
CT Scan	Yes, No, Unknown, Not Done
Total number of CT Scans during diagnosis	numeric
Total number of CT Scans during treatment	numeric
Total Number of post-treatment CT scans	numeric
Bronchoscopy	Yes, No, Unknown, Not Done
Date of Initial Culture	(mm/dd/yyyy)
Baseline culture	Positive, Negative, Unknown, Not Done
Induced Sputum Procedure	Yes, No, Unknown, Not Done
Number of Cultures, Induced, during diagnosis	numeric
Number of Cultures, not Induced, during diagnosis	numeric
Total Number of Induced Sputum Procedure(s)	numeric
Overall Number of Cultures Ordered	numeric
Time to Culture Conversion (in days from treatment start)	numeric
Date of Initial Smear	(mm/dd/yyyy)
AFB Smear	Positive, Negative, Unknown, Not Done
Number of AFB Smears during diagnosis	numeric
Number of AFB Smears during treatment	numeric
Number of ECGs	numeric
Number of audiometric tests	numeric
Anti-HBc	Positive, Negative, Unknown, Not Done
Ag HBs	Positive, Negative, Unknown, Not Done
Anti-HBb	Positive, Negative, Unknown, Not Done
Hepatitis Blood Draws	numeric
HbA1c	numeric
Site of TB	Pulmonary, Extrapulmonary
Site of EPTB	character
Number of Ultrasounds of abdomen	numeric

Variable	Value
Number of Biopsy: endometrium	numeric
Number of X-Rays: chest lordotic view only	numeric
Number of X-Rays: Cspine (4 views or more)	numeric
Number of MRI: C and T spine C-	numeric
Number of MRI: total spine C-T-L C+	numeric
Number of MRI: head C- C+	numeric
Number of CT Scans: T+ L spine C-	numeric
Number of Puncture: lumbar	numeric
Number of Puncture: other	numeric
Number of Ultrasounds: doppler other	numeric
Number of Ultrasounds: face or neck/ thyroid/ parathy	numeric
Number of Ultrasounds: doppler abdominal/ pelvis	numeric
Number of Ultrasounds: pelvis TVS or TAS	numeric
Number of Biopsy: lymph node	numeric
Number of Biopsy: vertebral	numeric
Number of X-Rays: chest 2 views + lordotic view	numeric
Number of MRI: total spine C-T-L C+	numeric
Number of MRI: head C- C+	numeric
Number of CT Scans: abdomen and pelvis	numeric
Number of Microbiology: tissue bacterial culture	numeric
Number of Microbiology: fungus/deep culture	numeric
Initial Tuberculin Skin Test Date	(mm/dd/yyyy)
Tuberculin Skin Test Result	Positive, Negative, Unknown
Overall number of tuberculin skin test	numeric
Initial Interferon-Gamma Release Assay Date	(mm/dd/yyyy)
Type of Interferon-Gamma Release Assay	QuantiFERON, T-SPOT.TB
Interferon-Gamma Release Assay Result	Positive, Negative, Unknown
Overall number of Interferon-Gamma Release Assay	Numeric
Initial LPA First-Line Specimen Date	(mm/dd/yyyy)
LPA - Isoniazid Resistant	Yes, No, Unknown
LPA - Rifampin Resistant	Yes, No, Unknown
Overall number of First-Line LPA ordered	numeric
Initial Xpert Date	(mm/dd/yyyy)
Xpert Result	resistant, susceptible, unknown
Overall number of Xpert Ordered	numeric
First-Line Phenotypic DST Specimen Date	(mm/dd/yyyy)
First-Line Phenotypic DST Results Date	(mm/dd/yyyy)
First-Line Phenotypic DST Results Reception Date	(mm/dd/yyyy)
Resistance to isoniazid	resistant, susceptible, unknown
Resistance to rifampin	resistant, susceptible, unknown
Resistance to ethambutol	resistant, susceptible, unknown
Resistance to pyrazinamide	resistant, susceptible, unknown
Resistance to rifabutin	resistant, susceptible, unknown
Overall Number of First-line phenotypic DST ordered	numeric
Resistance to amikacin	resistant, susceptible, unknown
Resistance to kanamycin	resistant, susceptible, unknown
Resistance to capreomycin	resistant, susceptible, unknown
Resistance to ofloxacin	resistant, susceptible, unknown
Resistance to levofloxacin	resistant, susceptible, unknown
Resistance to moxifloxacin	resistant, susceptible, unknown
Resistance to ciprofloxacin	resistant, susceptible, unknown
Resistance to gatifloxacin	resistant, susceptible, unknown
Resistance to clofazimine	resistant, susceptible, unknown
Resistance to ethionamide	resistant, susceptible, unknown
Resistance to cycloserine	resistant, susceptible, unknown
Resistance to linezolid	resistant, susceptible, unknown
Resistance to streptomycin	resistant, susceptible, unknown
Resistance to PAS	resistant, susceptible, unknown
Resistance to amoxicillin-clavulanate	resistant, susceptible, unknown
Resistance to imipenem-cilastatin	resistant, susceptible, unknown
Resistance to meropenem	resistant, susceptible, unknown
Resistance to clarithromycin	resistant, susceptible, unknown
Resistance to azithromycin	resistant, susceptible, unknown
Resistance to bedaquiline	resistant, susceptible, unknown
Resistance to delamanid	resistant, susceptible, unknown
Overall Number of Second-line phenotypic DST ordered	numeric
Hospitalized at Treatment Start?	Yes, No
Duration of Initial Hospitalization (days)	numeric

Variable	Value
Hospitalized During Treatment?	Yes, No
Reason Hospitalized	Adverse Event, Failure, Non-Adherence, Other
Length Hospitalized	numeric
Received isoniazid?	Yes, No
Record dose, start and stop date, method of administration, frequency of administration	Various
Received rifampin?	Yes, No
Record dose, start and stop date, method of administration, frequency of administration	Various
Received ethambutol?	Yes, No
Record dose, start and stop date, method of administration, frequency of administration	Various
Received pyrazinamide?	Yes, No
Record dose, start and stop date, method of administration, frequency of administration	Various
Received rifabutin?	Yes, No
Record dose, start and stop date, method of administration, frequency of administration	Various
Received rifapentine?	Yes, No
Record dose, start and stop date, method of administration, frequency of administration	Various
Received streptomycin?	Yes, No
Record dose, start and stop date, method of administration, frequency of administration	Various
Received capreomycin?	Yes, No
Record dose, start and stop date, method of administration, frequency of administration	Various
Received kanamycin?	Yes, No
Record dose, start and stop date, method of administration, frequency of administration	Various
Received amikacin?	Yes, No
Record dose, start and stop date, method of administration, frequency of administration	Various
Received ofloxacin?	Yes, No
Record dose, start and stop date, method of administration, frequency of administration	Various
Received ciprofloxacin?	Yes, No
Record dose, start and stop date, method of administration, frequency of administration	Various
Received moxifloxacin?	Yes, No
Record dose, start and stop date, method of administration, frequency of administration	Various
Received levofloxacin?	Yes, No
Record dose, start and stop date, method of administration, frequency of administration	Various
Received gatifloxacin?	Yes, No
Record dose, start and stop date, method of administration, frequency of administration	Various
Received linezolid?	Yes, No
Record dose, start and stop date, method of administration, frequency of administration	Various
Received ethionamide?	Yes, No
Record dose, start and stop date, method of administration, frequency of administration	Various
Received prothionamide?	Yes, No
Record dose, start and stop date, method of administration, frequency of administration	Various
Received PAS?	Yes, No
Record dose, start and stop date, method of administration, frequency of administration	Various
Received cycloserine?	Yes, No
Record dose, start and stop date, method of administration, frequency of administration	Various
Received terizidone?	Yes, No
Record dose, start and stop date, method of administration, frequency of administration	Various
Received clofazimine?	Yes, No
Record dose, start and stop date, method of administration, frequency of administration	Various

Variable	Value
Received imipenem-cilastatin?	Yes, No
Record dose, start and stop date, method of administration, frequency of administration	Various
Received meropenem?	Yes, No
Record dose, start and stop date, method of administration, frequency of administration	Various
Received clarithromycin?	Yes, No
Record dose, start and stop date, method of administration, frequency of administration	Various
Received amoxicillin-clavulanate?	Yes, No
Record dose, start and stop date, method of administration, frequency of administration	Various
Received azithromycin?	Yes, No
Record dose, start and stop date, method of administration, frequency of administration	Various
Received bedaquiline?	Yes, No
Record dose, start and stop date, method of administration, frequency of administration	Various
Received delamanid?	Yes, No
Record dose, start and stop date, method of administration, frequency of administration	Various
Received other TB medication?	Yes, No
Record dose, start and stop date, method of administration, frequency of administration	Various
PICC Line?	Yes, No
Number of PICC Line Insertions	numeric
Given B6?	Yes, No
Adjunctive non-TB Med Given?	Yes, No
Record dose, start and stop date, method of administration, frequency of administration	Various
AST Measurements	numeric
ALP Measurements	numeric
ALT Measurements	numeric
Creatinine Measurements	numeric
Bilirubin Measurements	numeric
CBC Measurements	numeric
Albumin Measurements	numeric
TSH Measurements	numeric
Mg Measurements	numeric
Urea Measurements	numeric
Calcium Measurements	numeric
Phosphate Measurements	numeric
Sodium Measurements	numeric
Potassium Measurements	numeric
Chloride Measurements	numeric
Amikacin Serum Measurements	numeric
Other serum drug level measurements	numeric
Liver transaminase panel measurements (not included elsewhere)	numeric
Number of blood draws	numeric
Therapeutic drug monitoring done	Yes, No
Number of TDM tests sent out	numeric
Incentives Given?	Yes, No
Type of Incentive	character
Value of Incentive	numeric
Case manager?	Yes, No
Number of Contacts Investigated - HOME (PRIMARY)	numeric
Number of Contacts Investigated - HOME (SECONDARY)	numeric
Number of Contacts Investigated - SOCIAL (PRIMARY)	numeric
Number of Contacts Investigated - SOCIAL (SECONDARY)	numeric
Number of Contacts Investigated - SCHOOL & WORK (PRIMARY)	numeric
Number of Contacts Investigated - SCHOOL & WORK (SECONDARY)	numeric
Number of Contacts Investigated - TRAVEL (PRIMARY)	numeric

Variable	Value
Number of Contacts Investigated - TRAVEL (SECONDARY)	numeric
Number of Contacts Investigated - UNASIGNED & OTHER	numeric
No. of Emergency Department Visits	numeric
Clinic Visits (Doctor); stratified diagnosis, treatment, post-treatment	numeric
Clinic Visits (Resident); stratified diagnosis, treatment, post-treatment	numeric
Clinic Visits (Internal Medicine); stratified diagnosis, treatment, post-treatment	numeric
Clinic Visits (Specialist) - Psychiatry; stratified diagnosis, treatment, post-treatment	numeric
Clinic Visits (Specialist) - Ophthalmologist; stratified diagnosis, treatment, post-treatment	numeric
Clinic Visits (Specialist) - ENT; stratified diagnosis, treatment, post-treatment	numeric
Physician (Specialist) - Neurologist; stratified diagnosis, treatment, post-treatment	numeric
Clinic Visits (Specialist) - Gastroenterologist; stratified diagnosis, treatment, post-treatment	numeric
Clinic Visits (Specialist) - Dermatologist; stratified diagnosis, treatment, post-treatment	numeric
Clinic Visits (Specialist) - Hematologist; stratified diagnosis, treatment, post-treatment	numeric
Clinic Visits (Specialist) - Nutritionist; stratified diagnosis, treatment, post-treatment	numeric
Nurse Phone Calls; stratified diagnosis, treatment, post-treatment	numeric
MD Phone Calls; stratified diagnosis, treatment, post-treatment	numeric
Overall Number of Patient Visits to Clinic; stratified diagnosis, treatment, post-treatment	numeric
Number of Home Visits; stratified diagnosis, treatment, post-treatment	numeric
Number of Times an Interpreter was Used; stratified diagnosis, treatment, post-treatment	numeric
Social worker visits; stratified diagnosis, treatment, post-treatment	numeric
Overall DOT Visits	numeric
Number of Pharmacy DOT Visits	numeric
Number of Clinic DOT Visits	numeric
Number of Home DOT Visits	numeric
Isolated?	Yes, No, Unknown
Isolation Start Date	(mm/dd/yyyy)
Isolation Stop Date	(mm/dd/yyyy)
Medication Discontinued?	Yes, No
Drug Stopped	character
Reason Drug Discontinued	character
Type of AE	character
Did Patient Start Alternate TB Therapy?	Yes, No
Specify New TB Therapy	character
Final Treatment Outcome	Complete, Death, Failure, Adverse Event, Lost to Follow-up
Date of Final Outcome	(mm/dd/yyyy)
Duration of Treatment (days)	numeric
Adherence	At Least 80%, Less Than 80%
If Died, Related to TB?	Yes, No
Were Post-Treatment Monitoring Services Provided?	Yes, No, Unknown
Duration of Post-Treatment Monitoring (days)	numeric
Did Patient Experience Recurrence?	Yes, No, Unknown
Date of Recurrence	(mm/dd/yyyy)

Abbreviations: TB, tuberculosis; LPA, line probe assay; DOT, directly observed therapy; MD, medical doctor; AE, adverse event; DST, drug susceptibility test; AFB, acid-fast bacilli; ART, antiretroviral therapy

Appendix Table 2. Data Fields for Costs Considered

Item	Comment (if necessary)
Inpatient Costs	
Per day in the hospital	
Physician visit	
Resident visit	
Psychiatry visit	
Nutritionist visit	
Ophthalmologist visit	
Neurologist visit	
Gastroenterologist visit	
Hematologist visit	
Other Costs (Diagnosis, Treatment, Post-Treatment, Public Health)	
Tuberculin Skin Test	
Interferon-Gamma Release Assay	
HIV Test	Missing from Ontario
Chest X-Ray	
Smear (for TB)	
Culture (for TB)	
Sputum Induction	
First Line DST	
Second Line DST	
First Line LPA	
Second Line LPA	
Xpert MTB/RIF	
Ultrasound (various)	
Biopsy (various)	
MRI (various) costs	
CT scan (various) costs	
Fungal Culture	
Bacterial Culture	
Lumbar Puncture	
Bronchoscopy	
HBA1c	
Hepatitis B Serology	
Hepatitis C Serology	
Blood Draw Cost	
Initial Physician Consultation	
Initial Ophthalmology Consultation	
Initial ENT Consultation	
Initial Gastroenterologist Consultation	
Initial Dermatologist Consultation	
Initial Hematology Consultation	
Nutritionist Visit	
Chest X-Ray Interpretation	
Interpreter	
Isoniazid	Specify dose and cost for formulation
Rifampin	Specify dose and cost for formulation
Pyrazinamide	Specify dose and cost for formulation
Ethambutol	Specify dose and cost for formulation
Rifapentine	Specify dose and cost for formulation
Rifabutin	Specify dose and cost for formulation
Streptomycin	Specify dose and cost for formulation
Amikacin	Specify dose and cost for formulation
Capreomycin	Specify dose and cost for formulation
Kanamycin	Specify dose and cost for formulation
Moxifloxacin	Specify dose and cost for formulation
Levofloxacin	Specify dose and cost for formulation
Gatifloxacin	Specify dose and cost for formulation
Ofloxacin	Specify dose and cost for formulation
Ciprofloxacin	Specify dose and cost for formulation
Linezolid	Specify dose and cost for formulation
Clofazimine	Specify dose and cost for formulation
Ethionamide	Specify dose and cost for formulation; cost only available in Quebec
Prothionamide	Specify dose and cost for formulation
Cycloserine	Specify dose and cost for formulation
Terizidone	Specify dose and cost for formulation
Bedaquiline	Specify dose and cost for formulation

Item	Comment (if necessary)
Delamanid	Specify dose and cost for formulation
Imipenem-Cilastatin	Specify dose and cost for formulation
Meropenem	Specify dose and cost for formulation
Amoxicillin-Clavulanate	Specify dose and cost for formulation
Clarithromycin	Specify dose and cost for formulation
Azithromycin	Specify dose and cost for formulation
PAS	Specify dose and cost for formulation
Other TB Medication or Adjunct Drug Given	Specify type of drug, dose, and cost
AST	
ALP	
ALT	
Creatinine	
Bilirubin	
CBC	
Sodium	
Potassium	
Chloride	
Calcium	
Magnesium	
Phosphate	
Albumin	
Urea	
TSH	
Amikacin Drug Levels	Missing for Ontario
Drug Levels for Other Drugs	
ECG Measurement	
Audiometry Assessment	
Follow-up Physician Visit	
Follow-up Ophthalmology Visit	
Follow-up ENT Visit	
Follow-up Gastroenterologist Visit	
Follow-up Dermatologist Visit	
Follow-up Hematology Visit	
Nurse Home Visit	Missing for Ontario
Social Worker Visit	
Home Visit for DOT	Per visit; microcosting information missing for Ontario—used top-down approach
Clinic Visit for DOT	Per visit; microcosting information missing for Ontario—used top-down approach
Pharmacy Visit DOT	Per visit; microcosting information missing for Ontario—used top-down approach
Contact Investigation	Per contact; only available in Ontario
Incentives	
Emergency Room Visit	

Abbreviations: TB, tuberculosis; LPA, line probe assay; DOT, directly observed therapy; MD, medical doctor; AE, adverse event; DST, drug susceptibility test; AFB, acid-fast bacilli; ART, antiretroviral therapy.

Appendix Table 3. Predictors Considered in Regression Analyses

All Forms of TB	TB Infection	DS-TB	INHR-TB	MDR-TB
Type of TB	Age (<40y vs. ≥40y)	Age (<40y vs. ≥40y)	Age (<40y vs. ≥40y)	Age (<40y vs. ≥40y)
Age (<40y vs. ≥40y)	Sex	Sex	Sex	Sex
Sex	Adverse Events (0 vs. ≥1)	Adverse Events (0 vs. ≥1)	Adverse Events (0 vs. ≥1)	Adverse Events (0 vs. ≥1)
Adverse Events (0 vs. ≥1)	HIV	HIV	HIV	HIV
HIV	Diabetes	Diabetes	Diabetes	Diabetes
Diabetes	Starting Regimen (Rifampin vs. Other)	Hospitalization (<2mo vs. ≥2mo)	Hospitalization (<2mo vs. ≥2mo)	Hospitalization (<2mo vs. ≥2mo)
		Sputum Smear	Sputum Smear	Sputum Smear
		Cavities on Chest X-Ray	Cavities on Chest X-Ray	Cavities on Chest X-Ray
		TB Location (Pulmonary vs. Extrapulmonary)	TB Location (Pulmonary vs. Extrapulmonary)	TB Location (Pulmonary vs. Extrapulmonary)
		Culture Conversion Time (<2mo vs. ≥2mo)	Culture Conversion Time (<2mo vs. ≥2mo)	Culture Conversion Time (<2mo vs. ≥2mo)
		Number of Contacts	Number of Contacts	Number of Contacts
		Received DOT	Received DOT	Received DOT
				Resistance (Susceptible to both Fluoroquinolone and SLI vs. Not)

Abbreviations: TB, tuberculosis; DS-TB, drug-susceptible tuberculosis; INHR-TB, isoniazid-resistant tuberculosis; MDR-TB, multidrug-resistant tuberculosis; DOT, directly observed therapy; SLI, second-line injectable

Appendix Table 4. Median Costs for Each Form of Tuberculosis Stratified by Demographic Characteristics and Treatment Outcomes

Group Comparison	TB Infection	DS-TB	INHR-TB	MDR-TB
Age <40 Years	n = 53	n = 41	n = 30	n = 36
Median (IQR) Total Costs	$694 (IQR: $574 to $1101)	$11,299 (IQR: $4089 to $25,435)	$15,287 (IQR: $7029 to $35,608)	$119,531 (IQR: $87,476 to $169,970)
Median (IQR) Cost of Diagnosis	$252 (IQR: $194 to $367)	$714 (IQR: $488 to $1001)	$786 (IQR: $688 to $1051)	$1083 (IQR: $974 to $1334)
Median (IQR) Cost of Treatment	$437 (IQR: $350 to $710)	$2116 (IQR: $1615 to $2615)	$2685 (IQR: $2110 to $3716)	$68,870 (IQR: $35,063 to $117,520)
Median (IQR) Cost of Post-Treatment Monitoring	$0 (IQR: $0 to $0)	$137 (IQR: $0 to $352)	$109 (IQR: $70 to $151)	$174 (IQR: $54 to $326)
Median (IQR) Cost of Hospitalization	$0 (IQR: $0 to $0)	$0 (IQR: $0 to $15,600)	$4128 (IQR: $0 to $13,812)	$41,781 (IQR: $35,075 to $53,811)
Median (IQR) Cost for Public Health Interventions	$0 (IQR: $0 to $0)	$2308 (IQR: $577 to $5232)	$5028 (IQR: $1731 to $6212)	$6490 (IQR: $4655 to $6864)
Age ≥40 Years	n = 37	n = 49	n = 41	n = 26
Median (IQR) Total Costs	$870 (IQR: $690 to $1350)	$12,441 (IQR: $6698 to $23,062)	$23,461 (IQR: $7341 to $41,396)	$110981 (IQR: $72600 to $150,573)
Median (IQR) Cost of Diagnosis	$274 (IQR: $217 to $414)	$684 (IQR: $604 to $1034)	$828 (IQR: $618 to $1043)	$1042 (IQR: $925 to $1313)
Median (IQR) Cost of Treatment	$589 (IQR: $427 to $808)	$2150 (IQR: $1585 to $3423)	$3226 (IQR: $2345 to $4040)	$45,853 (IQR: $21,294 to $96,920)
Median (IQR) Cost of Post-Treatment Monitoring	$0 (IQR: $0 to $0)	$141 (IQR: $54 to $277)	$145 (IQR: $26 to $274)	$220 (IQR: $16 to $367)
Median (IQR) Cost of Hospitalization	$0 (IQR: $0 to $0)	$3900 (IQR: $0 to $14,950)	$13,000 (IQR: $0 to $31,856)	$40,815 (IQR: $35,542 to $61,413)
Median (IQR) Cost for Public Health Interventions	$0 (IQR: $0 to $0)	$3462 (IQR: $697 to $5232)	$2885 (IQR: $581 to $6115)	$6378 (IQR: $4657 to $6754)
Female Sex	n = 55	n = 50	n = 38	n = 34
Median (IQR) Total Costs	$853 (IQR: $592 to $1333)	$13,831 (IQR: $5203 to $27,262)	$14,907 (IQR: $6767 to $33,751)	$116,751 (IQR: $87,240 to $178,390)
Median (IQR) Cost of Diagnosis	$315 (IQR: $217 to $362)	$737 (IQR: $600 to $1112)	$709 (IQR: $581 to $994)	$1015 (IQR: $894 to $1322)
Median (IQR) Cost of Treatment	$550 (IQR: $385 to $818)	$2203 (IQR: $1693 to $3249)	$2490 (IQR: $2077 to $3898)	$59,343 (IQR: $30,591 to $111,895)
Median (IQR) Cost of Post-Treatment Monitoring	$0 (IQR: $0 to $18)	$139 (IQR: $53 to $282)	$120 (IQR: $47 to $177)	$270 (IQR: $71 to $399)
Median (IQR) Cost of Hospitalization	$0 (IQR: $0 to $0)	$0 (IQR: $0 to $18,095)	$9750 (IQR: $0 to $21,775)	$45,654 (IQR: $35,822 to $62,650)
Median (IQR) Cost for Public Health Interventions	$0 (IQR: $0 to $0)	$2885 (IQR: $1154 to $5353)	$2596 (IQR: $1154 to $5702)	$6390 (IQR: $4736 to $6876)
Male Sex	n = 35	n = 40	n = 33	n = 28
Median (IQR) Total Costs	$748 (IQR: $587 to $1008)	$11,533 (IQR: $3678 to $17,340)	$24,279 (IQR: $8997 to $43,494)	$119,051 (IQR: $76,174 to $151,917)
Median (IQR) Cost of Diagnosis	$217 (IQR: $216 to $380)	$684 (IQR: $498 to $911)	$883 (IQR: $785 to $1119)	$1093 (IQR: $1052 to $1331)
Median (IQR) Cost of Treatment	$476 (IQR: $370 to $659)	$2042 (IQR: $1559 to $2679)	$3225 (IQR: $2496 to $3936)	$62,567 (IQR: $30,105 to $101,609)
Median (IQR) Cost of Post-Treatment Monitoring	$0 (IQR: $0 to $0)	$139 (IQR: $0 to $283)	$130 (IQR: $26 to $224)	$145 (IQR: $0 to $276)
Median (IQR) Cost of Hospitalization	$0 (IQR: $0 to $0)	$3250 (IQR: $0 to $8775)	$11,050 (IQR: $0 to $31,054)	$39,476 (IQR: $33,962 to $50,457)
Median (IQR) Cost for Public Health Interventions	$0 (IQR: $0 to $0)	$3462 (IQR: $542 to $5224)	$5440 (IQR: $1094 to $6220)	$6409 (IQR: $4163 to $6728)
No Drug Stopped due to Adverse Event	n = 83	n = 52	n = 42	n = 10
Median (IQR) Total Costs	$781 (IQR: $587 to $1194)	$7101 (IQR: $3678 to $18,584)	$13,422 (IQR: $5297 to $33,751)	$97,683 (IQR: $79,455 to $124,357)
Median (IQR) Cost of Diagnosis	$252 (IQR: $204 to $345)	$701 (IQR: $498 to $981)	$818 (IQR: $659 to $1003)	$1083 (IQR: $1074 to $1098)
Median (IQR) Cost of Treatment	$496 (IQR: $374 to $757)	$2044 (IQR: $1559 to $2617)	$2556 (IQR: $2091 to $3274)	$52,304 (IQR: $30,894 to $100,856)
Median (IQR) Cost of Post-Treatment Monitoring	$0 (IQR: $0 to $0)	$139 (IQR: $48 to $327)	$121 (IQR: $12 to $177)	$79 (IQR: $62 to $182)
Median (IQR) Cost of Hospitalization	$0 (IQR: $0 to $0)	$0 (IQR: $0 to $8450)	$2002 (IQR: $0 to $15,337)	$37,671 (IQR: $34,764 to $41,731)
Median (IQR) Cost for Public Health Interventions	$0 (IQR: $0 to $0)	$2308 (IQR: $542 to $5218)	$2596 (IQR: $1154 to $6029)	$6365 (IQR: $2277 to $6628)

Group Comparison	TB Infection	DS-TB	INHR-TB	MDR-TB
At Least One Drug Stopped due to Adverse Event	n = 7	n = 38	n = 29	n = 52
Median (IQR) Total Costs	$1037 (IQR: $959 to $1230)	$14,099 (IQR: $7921 to $27,465)	$24,585 (IQR: $13,255 to $51,242)	$121,660 (IQR: $82,641 to $166,115)
Median (IQR) Cost of Diagnosis	$367 (IQR: $336 to $424)	$718 (IQR: $594 to $1064)	$857 (IQR: $661 to $1054)	$1083 (IQR: $892 to $1334)
Median (IQR) Cost of Treatment	$710 (IQR: $550 to $774)	$2408 (IQR: $1922 to $3405)	$3613 (IQR: $2448 to $4474)	$61,426 (IQR: $30,004 to $111,807)
Median (IQR) Cost of Post-Treatment Monitoring	$0 (IQR: $0 to $9)	$128 (IQR: $0 to $253)	$133 (IQR: $66 to $274)	$237 (IQR: $0 to $357)
Median (IQR) Cost of Hospitalization	$0 (IQR: $0 to $0)	$5525 (IQR: $0 to $19,339)	$15,028 (IQR: $4550 to $37,470)	$43,486 (IQR: $35,334 to $60,823)
Median (IQR) Cost for Public Health Interventions	$0 (IQR: $0 to $0)	$4039 (IQR: $1298 to $5695)	$5193 (IQR: $581 to $6218)	$6437 (IQR: $4740 to $6860)
Completed Treatment	n = 77	n = 83	n = 63	n = 49
Median (IQR) Total Costs	$851 (IQR: $596 to $1244)	$11,855 (IQR: $4259 to $25,450)	$22,110 (IQR: $7390 to $42,445)	$125,978 (IQR: $86,108 to $165,586)
Median (IQR) Cost of Diagnosis	$252 (IQR: $194 to $393)	$694 (IQR: $513 to $1017)	$817 (IQR: $657 to $1002)	$1083 (IQR: $926 to $1331)
Median (IQR) Cost of Treatment	$555 (IQR: $392 to $784)	$2157 (IQR: $1656 to $3242)	$3063 (IQR: $2362 to $4052)	$63,096 (IQR: $34,455 to $112,913)
Median (IQR) Cost of Post-Treatment Monitoring	$0 (IQR: $0 to $18)	$141 (IQR: $53 to $297)	$132 (IQR: $88 to $210)	$220 (IQR: $64 to $320)
Median (IQR) Cost of Hospitalization	$0 (IQR: $0 to $0)	$0 (IQR: $0 to $16250)	$11,050 (IQR: $0 to $31,528)	$40,952 (IQR: $35,412 to $54,112)
Median (IQR) Cost for Public Health Interventions	$0 (IQR: $0 to $0)	$2885 (IQR: $577 to $5232)	$3096 (IQR: $1141 to $6188)	$6570 (IQR: $5557 to $6901)
Did Not Complete Treatment	n = 13	n = 7	n = 8	n = 13
Median (IQR) Total Costs	$549 (IQR: $368 to $818)	$12,441 (IQR: $10104 to $18,574)	$11,480 (IQR: $6255 to $16,632)	$85,770 (IQR: $71,565 to $118,534)
Median (IQR) Cost of Diagnosis	$327 (IQR: $217 to $337)	$831 (IQR: $654 to $982)	$1057 (IQR: $727 to $1104)	$1077 (IQR: $924 to $1224)
Median (IQR) Cost of Treatment	$211 (IQR: $150 to $481)	$1188 (IQR: $303 to $2168)	$1512 (IQR: $1319 to $1871)	$36,121 (IQR: $16,585 to $71,601)
Median (IQR) Cost of Post-Treatment Monitoring	$0 (IQR: $0 to $0)	$0 (IQR: $0 to $0)	$0 (IQR: $0 to $47)	$0 (IQR: $0 to $399)
Median (IQR) Cost of Hospitalization	$0 (IQR: $0 to $0)	$3900 (IQR: $1950 to $10,400)	$1300 (IQR: $0 to $11,212)	$41,480 (IQR: $35,100 to $61,708)
Median (IQR) Cost for Public Health Interventions	$0 (IQR: $0 to $0)	$4874 (IQR: $2020 to $5908)	$1154 (IQR: $577 to $3091)	$5096 (IQR: $4616 to $6071)

Abbreviations: IQR, interquartile range; TB, tuberculosis; DS-TB, drug-susceptible tuberculosis; INHR-TB, isoniazid-resistant tuberculosis; MDR-TB, multidrug-resistant tuberculosis

Appendix Table 5. Mean Costs for Each Form of Tuberculosis Stratified by Demographic Characteristics and Treatment Outcomes

Group Comparison	TB Infection	DS-TB	INHR-TB	MDR-TB
Age <40 Years	n = 53	n = 41	n = 30	n = 36
Mean Total Costs	$842	$14,875	$36,138	$142,532
Mean Cost of Diagnosis	$289	$774	$879	$1,236
Mean Cost of Treatment	$533	$2,459	$4,480	$83,650
Mean Cost of Post-Treatment Monitoring	$20	$189	$124	$226
Mean Cost of Hospitalization	$0	$7,553	$20,674	$49,508
Mean Cost for Public Health Interventions	$0	$3,900	$9,980	$7,913
Age ≥40 Years	n = 37	n = 49	n = 41	n = 26
Mean Total Costs	$1,024	$16,523	$29,567	$116,893
Mean Cost of Diagnosis	$335	$802	$846	$1,229
Mean Cost of Treatment	$665	$2,691	$4,759	$62,331
Mean Cost of Post-Treatment Monitoring	$24	$173	$197	$267
Mean Cost of Hospitalization	$0	$9,452	$19,443	$47,798
Mean Cost for Public Health Interventions	$0	$3,405	$4,322	$5,269
Female Sex	n = 55	n = 50	n = 38	n = 34
Mean Total Costs	$962	$16,827	$32,564	$144,151
Mean Cost of Diagnosis	$317	$845	$781	$1,158
Mean Cost of Treatment	$619	$2,631	$5,511	$82,182
Mean Cost of Post-Treatment Monitoring	$26	$185	$161	$295
Mean Cost of Hospitalization	$0	$9,333	$22,541	$53,881
Mean Cost for Public Health Interventions	$0	$3,832	$3,570	$6,635
Male Sex	n = 35	n = 40	n = 33	n = 28
Mean Total Costs	$846	$14,454	$32,089	$116,759
Mean Cost of Diagnosis	$293	$719	$951	$1,324
Mean Cost of Treatment	$537	$2,528	$3,640	$65,636
Mean Cost of Post-Treatment Monitoring	$16	$175	$172	$180
Mean Cost of Hospitalization	$0	$7,655	$16,994	$42,609
Mean Cost for Public Health Interventions	$0	$3,378	$10,332	$7,010
No Drug Stopped due to Adverse Event	n = 83	n = 52	n = 42	n = 10
Mean Total Costs	$901	$13,258	$30,559	$106,475
Mean Cost of Diagnosis	$301	$770	$860	$1,300
Mean Cost of Treatment	$577	$2,313	$4,040	$61,450
Mean Cost of Post-Treatment Monitoring	$22	$202	$149	$139
Mean Cost of Hospitalization	$0	$6,624	$17,900	$38,669
Mean Cost for Public Health Interventions	$0	$3,349	$7,610	$4,916
At Least One Drug Stopped due to Adverse Event	n = 7	n = 38	n = 29	n = 52
Mean Total Costs	$1,105	$19,212	$34,926	$136,647
Mean Cost of Diagnosis	$386	$815	$859	$1,220
Mean Cost of Treatment	$703	$2,957	$5,512	$77,260
Mean Cost of Post-Treatment Monitoring	$17	$151	$190	$263
Mean Cost of Hospitalization	$0	$11,274	$22,951	$50,737
Mean Cost for Public Health Interventions	$0	$4,015	$5,415	$7,168
Completed Treatment	n = 77	n = 83	n = 63	n = 49
Mean Total Costs	$964	$15,810	$34,395	$139,645
Mean Cost of Diagnosis	$309	$787	$851	$1,245
Mean Cost of Treatment	$630	$2,692	$5,023	$80,927
Mean Cost of Post-Treatment Monitoring	$24	$193	$182	$247
Mean Cost of Hospitalization	$0	$8,585	$21,827	$49,795
Mean Cost for Public Health Interventions	$0	$3,553	$6,512	$7,430
Did Not Complete Treatment	n = 13	n = 7	n = 8	n = 13
Mean Total Costs	$638	$15,327	$16,183	$102,137
Mean Cost of Diagnosis	$298	$815	$933	$1,186
Mean Cost of Treatment	$332	$1,318	$1,634	$51,276
Mean Cost of Post-Treatment Monitoring	$8	$37	$40	$226
Mean Cost of Hospitalization	$0	$8,608	$5,281	$45,004
Mean Cost for Public Health Interventions	$0	$4,549	$8,295	$4,445

Abbreviations: IQR, interquartile range; TB, tuberculosis; DS-TB, drug-susceptible tuberculosis; INHR-TB, isoniazid-resistant tuberculosis; MDR-TB, multidrug-resistant tuberculosis

Appendix Table 6. Median Costs for Each Form of Tuberculosis Stratified by Clinical Characteristics and Hospitalization

Group Comparison	DS-TB	INHR-TB	MDR-TB
No Hospitalization or Hospitalization <2 months	n = 85	n = 58	n = 16
Median (IQR) Total Costs	$11,394 (IQR: $4228 to $19,389)	$13,580 (IQR: $6193 to $24,923)	$97,215 (IQR: $54,858 to $140,209)
Median (IQR) Cost of Diagnosis	$694 (IQR: $518 to $1001)	$818 (IQR: $662 to $1041)	$996 (IQR: $729 to $1210)
Median (IQR) Cost of Treatment	$2116 (IQR: $1606 to $2910)	$2816 (IQR: $2211 to $3757)	$59,331 (IQR: $19,431 to $108,429)
Median (IQR) Cost of Post-Treatment Monitoring	$141 (IQR: $34 to $296)	$121 (IQR: $12 to $191)	$193 (IQR: $116 to $305)
Median (IQR) Cost of Hospitalization	$0 (IQR: $0 to $11,429)	$4128 (IQR: $0 to $13,975)	$24,621 (IQR: $17,790 to $31,355)
Median (IQR) Cost for Public Health Interventions	$2885 (IQR: $577 to $5311)	$2885 (IQR: $1134 to $5764)	$5109 (IQR: $654 to $6368)
Hospitalization ≥2 Months	n = 5	n = 13	n = 46
Median (IQR) Total Costs	$52,188 (IQR: $49,374 to $52,760)	$66,341 (IQR: $51,242 to $106,412)	$124,864 (IQR: $86,564 to $174,503)
Median (IQR) Cost of Diagnosis	$752 (IQR: $592 to $1192)	$871 (IQR: $654 to $1043)	$1083 (IQR: $996 to $1331)
Median (IQR) Cost of Treatment	$4176 (IQR: $3835 to $4621)	$4321 (IQR: $2791 to $4951)	$62,567 (IQR: $31,925 to $110,199)
Median (IQR) Cost of Post-Treatment Monitoring	$115 (IQR: $0 to $115)	$157 (IQR: $98 to $191)	$203 (IQR: $8 to $349)
Median (IQR) Cost of Hospitalization	$41,552 (IQR: $41,480 to $42,250)	$54,106 (IQR: $45,090 to $75,918)	$49,603 (IQR: $39,927 to $62,368)
Median (IQR) Cost for Public Health Interventions	$4616 (IQR: $3462 to $5193)	$6183 (IQR: $581 to $6220)	$6611 (IQR: $5429 to $6876)
Acid Fast Bacilli Smear Positive	n = 47	n = 35	n = 22
Median (IQR) Total Costs	$17,070 (IQR: $12,487 to $27,596)	$27,164 (IQR: $8286 to $56,992)	$156,251 (IQR: $91,590 to $194,886)
Median (IQR) Cost of Diagnosis	$640 (IQR: $510 to $923)	$779 (IQR: $671 to $1038)	$1011 (IQR: $817 to $1148)
Median (IQR) Cost of Treatment	$2571 (IQR: $1984 to $3857)	$3203 (IQR: $2362 to $4208)	$103,076 (IQR: $36,751 to $130,946)
Median (IQR) Cost of Post-Treatment Monitoring	$155 (IQR: $111 to $281)	$133 (IQR: $73 to $181)	$146 (IQR: $39 to $298)
Median (IQR) Cost of Hospitalization	$6500 (IQR: $0 to $19,175)	$11,700 (IQR: $0 to $35,310)	$48,097 (IQR: $39,626 to $54,012)
Median (IQR) Cost for Public Health Interventions	$5210 (IQR: $2885 to $5765)	$5497 (IQR: $2020 to $6218)	$6428 (IQR: $6144 to $6877)
Acid Fast Bacilli Smear Negative or Unknown	n = 43	n = 36	n = 40
Median (IQR) Total Costs	$5292 (IQR: $3099 to $10,292)	$14,820 (IQR: $5995 to $24,698)	$113,952 (IQR: $76,379 to $144,753)
Median (IQR) Cost of Diagnosis	$729 (IQR: $608 to $1113)	$836 (IQR: $646 to $1073)	$1102 (IQR: $958 to $1365)
Median (IQR) Cost of Treatment	$1883 (IQR: $1517 to $2248)	$2816 (IQR: $2079 to $3669)	$56,285 (IQR: $23,777 to $98,875)
Median (IQR) Cost of Post-Treatment Monitoring	$107 (IQR: $0 to $287)	$119 (IQR: $0 to $202)	$219 (IQR: $46 to $357)
Median (IQR) Cost of Hospitalization	$0 (IQR: $0 to $3250)	$8775 (IQR: $0 to $15,987)	$39,275 (IQR: $33,160 to $57,370)
Median (IQR) Cost for Public Health Interventions	$1154 (IQR: $353 to $2885)	$1731 (IQR: $577 to $5739)	$6393 (IQR: $1780 to $6755)
Cavities on Chest X-Ray	n = 30	n = 21	n = 15
Median (IQR) Total Costs	$16,574 (IQR: $12,464 to $27,138)	$25,795 (IQR: $7575 to $66,341)	$165,586 (IQR: $131,665 to $209,581)
Median (IQR) Cost of Diagnosis	$718 (IQR: $557 to $940)	$883 (IQR: $739 to $1119)	$1083 (IQR: $944 to $1275)
Median (IQR) Cost of Treatment	$2158 (IQR: $1813 to $3076)	$3203 (IQR: $2496 to $4407)	$107,991 (IQR: $74,093 to $127,872)
Median (IQR) Cost of Post-Treatment Monitoring	$179 (IQR: $109 to $385)	$158 (IQR: $114 to $224)	$309 (IQR: $79 to $374)
Median (IQR) Cost of Hospitalization	$7475 (IQR: $0 to $18,095)	$14,950 (IQR: $0 to $37,470)	$50,308 (IQR: $39,913 to $61,631)
Median (IQR) Cost for Public Health Interventions	$5219 (IQR: $3606 to $5767)	$5497 (IQR: $1154 to $6218)	$6456 (IQR: $6370 to $6817)
No Cavities on Chest X-Ray or Unknown	n = 60	n = 50	n = 47
Median (IQR) Total Costs	$7261 (IQR: $3995 to $19,379)	$17,412 (IQR: $6879 to $35,742)	$109,259 (IQR: $75,325 to $148,714)

Group Comparison	DS-TB	INHR-TB	MDR-TB
Median (IQR) Cost of Diagnosis	$701 (IQR: $515 to $1081)	$786 (IQR: $628 to $994)	$1083 (IQR: $925 to $1331)
Median (IQR) Cost of Treatment	$2071 (IQR: $1571 to $3178)	$2795 (IQR: $2077 to $3860)	$47,677 (IQR: $23,440 to $102,921)
Median (IQR) Cost of Post-Treatment Monitoring	$115 (IQR: $0 to $230)	$107 (IQR: $2 to $185)	$189 (IQR: $16 to $318)
Median (IQR) Cost of Hospitalization	$0 (IQR: $0 to $10,170)	$9750 (IQR: $0 to $17,020)	$39,876 (IQR: $32,658 to $53,912)
Median (IQR) Cost for Public Health Interventions	$1731 (IQR: $577 to $4920)	$2308 (IQR: $1102 to $6020)	$6380 (IQR: $2071 to $6791)
Disease Has No Extrapulmonary Involvement	n = 68	n = 51	n = 47
Median (IQR) Total Costs	$13,011 (IQR: $6108 to $23,655)	$21,322 (IQR: $7117 to $43,510)	$119,569 (IQR: $84,556 to $172,236)
Median (IQR) Cost of Diagnosis	$709 (IQR: $572 to $981)	$828 (IQR: $657 to $1055)	$1102 (IQR: $996 to $1377)
Median (IQR) Cost of Treatment	$2127 (IQR: $1615 to $3188)	$3024 (IQR: $2387 to $3919)	$62,038 (IQR: $26,708 to $108,560)
Median (IQR) Cost of Post-Treatment Monitoring	$137 (IQR: $34 to $287)	$132 (IQR: $73 to $206)	$222 (IQR: $46 to $348)
Median (IQR) Cost of Hospitalization	$3575 (IQR: $0 to $15,036)	$10,400 (IQR: $0 to $31,528)	$45,290 (IQR: $37,571 to $61,118)
Median (IQR) Cost for Public Health Interventions	$4745 (IQR: $1731 to $5450)	$5489 (IQR: $2020 to $6218)	$6600 (IQR: $6216 to $6868)
Disease Has Extrapulmonary Involvement	n = 22	n = 20	n = 15
Median (IQR) Total Costs	$5578 (IQR: $2988 to $25,163)	$17,444 (IQR: $7842 to $27,039)	$113,897 (IQR: $78,192 to $142,141)
Median (IQR) Cost of Diagnosis	$685 (IQR: $508 to $1129)	$757 (IQR: $666 to $952)	$969 (IQR: $856 to $1077)
Median (IQR) Cost of Treatment	$2240 (IQR: $1548 to $3121)	$2362 (IQR: $1971 to $3918)	$60,790 (IQR: $41,066 to $110,603)
Median (IQR) Cost of Post-Treatment Monitoring	$141 (IQR: $0 to $273)	$119 (IQR: $0 to $175)	$158 (IQR: $31 to $253)
Median (IQR) Cost of Hospitalization	$0 (IQR: $0 to $20,962)	$10,400 (IQR: $3088 to $15,987)	$35,412 (IQR: $24,621 to $41,781)
Median (IQR) Cost for Public Health Interventions	$577 (IQR: $238 to $1480)	$1141 (IQR: $399 to $1875)	$1486 (IQR: $1139 to $3812)

Abbreviations: IQR, interquartile range; DS-TB, drug-susceptible tuberculosis; INHR-TB, isoniazid-resistant tuberculosis; MDR-TB, multidrug-resistant tuberculosis

Appendix Table 7. Mean Costs for Each Form of Tuberculosis Stratified by Clinical Characteristics and Hospitalization

Group Comparison	DS-TB	INHR-TB	MDR-TB
No Hospitalization or Hospitalization <2 months	n = 85	n = 58	n = 16
Mean Total Costs	$13,741	$20,219	$98,227
Mean Cost of Diagnosis	$783	$854	$1,173
Mean Cost of Treatment	$2,504	$3,757	$68,994
Mean Cost of Post-Treatment Monitoring	$186	$159	$249
Mean Cost of Hospitalization	$6,669	$8,712	$23,236
Mean Cost for Public Health Interventions	$3,599	$6,736	$4,575
Hospitalization ≥2 Months	n = 5	n = 13	n = 46
Mean Total Costs	$50,300	$86,437	$143,451
Mean Cost of Diagnosis	$895	$884	$1,253
Mean Cost of Treatment	$3,968	$8,588	$76,698
Mean Cost of Post-Treatment Monitoring	$92	$196	$241
Mean Cost of Hospitalization	$41,186	$70,158	$57,679
Mean Cost for Public Health Interventions	$4,160	$6,611	$7,580
Acid Fast Bacilli Smear Positive	n = 47	n = 35	n = 22
Mean Total Costs	$21,486	$41,946	$166,159
Mean Cost of Diagnosis	$747	$834	$1,087
Mean Cost of Treatment	$2,889	$4,393	$101,149
Mean Cost of Post-Treatment Monitoring	$205	$181	$214
Mean Cost of Hospitalization	$12,397	$25,791	$54,209
Mean Cost for Public Health Interventions	$5,249	$10,747	$9,500
Acid Fast Bacilli Smear Negative or Unknown	n = 43	n = 36	n = 40
Mean Total Costs	$9,527	$23,007	$112,872
Mean Cost of Diagnosis	$835	$885	$1,313
Mean Cost of Treatment	$2,254	$4,883	$60,168
Mean Cost of Post-Treatment Monitoring	$154	$151	$259
Mean Cost of Hospitalization	$4,423	$14,297	$45,810
Mean Cost for Public Health Interventions	$1,861	$2,791	$5,322
Cavities on Chest X-Ray	n = 30	n = 21	n = 15
Mean Total Costs	$20,264	$45,617	$178,906
Mean Cost of Diagnosis	$773	$929	$1,255
Mean Cost of Treatment	$2,788	$3,407	$113,691
Mean Cost of Post-Treatment Monitoring	$247	$192	$306
Mean Cost of Hospitalization	$11,107	$29,561	$52,340
Mean Cost for Public Health Interventions	$5,349	$11,528	$11,315
No Cavities on Chest X-Ray or Unknown	n = 60	n = 50	n = 47
Mean Total Costs	$13,526	$26,768	$116,740
Mean Cost of Diagnosis	$798	$831	$1,226
Mean Cost of Treatment	$2,484	$5,160	$62,269
Mean Cost of Post-Treatment Monitoring	$148	$155	$223
Mean Cost of Hospitalization	$7,327	$15,932	$47,658
Mean Cost for Public Health Interventions	$2,771	$4,691	$5,365
Disease Has No Extrapulmonary Involvement	n = 68	n = 51	n = 47
Mean Total Costs	$16,293	$34,070	$137,823
Mean Cost of Diagnosis	$791	$864	$1,254
Mean Cost of Treatment	$2,597	$4,230	$75,683
Mean Cost of Post-Treatment Monitoring	$183	$172	$253
Mean Cost of Hospitalization	$8,421	$20,747	$52,771
Mean Cost for Public Health Interventions	$4,301	$8,057	$7,863
Disease Has Extrapulmonary Involvement	n = 22	n = 20	n = 15
Mean Total Costs	$14,161	$27,940	$112,846
Mean Cost of Diagnosis	$784	$849	$1,166
Mean Cost of Treatment	$2,549	$5,691	$71,660
Mean Cost of Post-Treatment Monitoring	$175	$150	$213
Mean Cost of Hospitalization	$9,099	$17,965	$36,319
Mean Cost for Public Health Interventions	$1,556	$3,286	$3,489

Abbreviations: DS-TB, drug-susceptible tuberculosis; INHR-TB, isoniazid-resistant tuberculosis; MDR-TB, multidrug-resistant tuberculosis

Appendix Table 8. Mean Costs of Different Forms of Tuberculosis

Group	Mean Total Cost	Mean Cost of Diagnosis	Mean Cost of Treatment	Mean Cost of Post-Treatment Monitoring	Mean Cost of Hospitalization	Mean Cost for Public Health Interventions
TB Infection						
British Columbia Centre for Disease Control (n=30)	$896	$342	$549	$5	$0	$0
West Park Healthcare Centre (n=30)	$1,207	$363	$791	$53	$0	$0
Montreal Chest Institute (n=30)	$647	$218	$421	$8	$0	$0
Isoniazid Only (n=49)	$1,055	$349	$678	$28	$0	$0
Rifampin Only (n=35)	$671	$236	$422	$13	$0	$0
Other Isoniazid-Containing Regimen (n=6)	$1,215	$385	$810	$19	$0	$0
DS-TB Disease						
British Columbia Centre for Disease Control (n=30)	$20,893	$945	$3,067	$162	$12,622	$4,097
West Park Healthcare Centre (n=30)	$15,591	$670	$1,934	$169	$8,928	$3,890
Montreal Chest Institute (n=30)	$10,833	$753	$2,754	$211	$4,211	$2,903
INHR-TB Disease						
British Columbia Centre for Disease Control (n=30)	$21,415	$912	$2,657	$124	$11,327	$6,393
West Park Healthcare Centre (n=27)	$52,090	$820	$7,698	$156	$38,338	$5,077
Montreal Chest Institute (n=14)	$17,679	$824	$2,998	$274	$3,030	$10,554
MDR-TB Disease						
British Columbia Centre for Disease Control (n=11)	$187,836	$1,229	$132,798	$386	$42,056	$11,365
West Park Healthcare Centre (n=45)	$120,152	$1,298	$61,196	$188	$52,032	$5,438
Montreal Chest Institute (n=6)	$116,225	$752	$69,569	$388	$36,827	$8,690
Susceptible to both Fluoroquinolones and Second-Line Injectables (n=50)	$118,643	$1,237	$67,591	$233	$43,082	$6,499
Resistant to a Fluoroquinolone and/or Second-Line Injectable (n=12)	$186,520	$1,216	$104,371	$282	$72,576	$8,075

Abbreviations: TB, tuberculosis; DS-TB, drug-susceptible tuberculosis; INHR-TB, isoniazid-resistant tuberculosis; MDR-TB, multidrug-resistant tuberculosis

Appendix Table 9. Univariable Analysis of Characteristics Associated with Increasing or Decreasing Costs, Reported as Cost Ratios and 95% Confidence Intervals

Characteristic	All Patients (n=313)	TB Infection (n=90)	DS-TB (n=90)	INHR-TB (n=71)	MDR-TB (n=62)
TB Type		--	--	--	--
DS-TB	1.0 (reference)	--	--	--	--
TB Infection	0.08 (0.06 to 0.1)	--	--	--	--
INHR-TB	1.64 (1.28 to 2.09)	--	--	--	--
MDR-TB	9.14 (7.04 to 11.88)	--	--	--	--
Age					
<40 years	1.0 (reference)	1.0 (reference)	1.0 (reference)	1.0 (reference)	1.0 (reference)
≥40 years	1.07 (0.69 to 1.68)	1.29 (1.09 to 1.53)	0.93 (0.59 to 1.46)	1.13 (0.73 to 1.76)	0.81 (0.62 to 1.07)
Sex					
Female	1.0 (reference)	1.0 (reference)	1.0 (reference)	1.0 (reference)	1.0 (reference)
Male	1.14 (0.75 to 1.73)	0.99 (0.83 to 1.17)	0.74 (0.49 to 1.11)	1.30 (0.85 to 2.00)	0.88 (0.67 to 1.17)
HIV					
HIV-Negative or unknown	1.0 (reference)	--*	1.0 (reference)	--*	1.0 (reference)
HIV-Positive	6.8 (0.55 to 84.13)	--*	5.74 (0.89 to 37.13)	--*	0.66 (0.23 to 1.93)
Diabetes					
No Diabetes or unknown	1.0 (reference)	1.0 (reference)	1.0 (reference)	1.0 (reference)	1.0 (reference)
Has Diabetes	1.15 (0.64 to 2.05)	1.1 (0.85 to 1.42)	1.32 (0.874 to 2.32)	1.63 (0.88 to 3.03)	0.88 (0.61 to 1.27)
Adverse Events					
None	1.0 (reference)	1.0 (reference)	1.0 (reference)	1.0 (reference)	1.0 (reference)
At Least One	6.97 (4.83 to 10.04)	0.99 (0.72 to 1.35)	1.56 (1.05 to 2.32)	1.50 (0.96 to 2.34)	1.11 (0.77 to 1.61)
Hospitalization	--	--			
None or <2 months	--	--	1.0 (reference)	1.0 (reference)	1.0 (reference)
≥2 months	--	--	4.74 (2.08 to 10.83)	3.98 (2.42 to 6.54)	1.75 (1.30 to 2.35)
Acid Fast Bacilli Smear	--	--			
Negative or unknown	--	--	1.0 (reference)	1.0 (reference)	1.0 (reference)
Positive	--	--	2.67 (1.87 to 3.82)	1.92 (1.28 to 2.89)	1.29 (0.98 to 1.71)
Cavities on Chest X-Ray	--	--			
None or unknown	--	--	1.0 (reference)	1.0 (reference)	1.0 (reference)
Yes	--	--	1.73 (1.15 to 2.61)	1.26 (0.78 to 2.02)	1.53 (1.13 to 2.07)
TB Location	--	--			
Pulmonary Only	--	--	1.0 (reference)	1.0 (reference)	1.0 (reference)
Extrapulmonary Involvement	--	--	0.61 (0.39 to 0.96)	0.99 (0.6 to 1.62)	0.90 (0.66 to 1.24)
Number of Contacts	--	--			
Per Additional Contact	--	--	1.07 (1.04 to 1.11)	1.02 (1.01 to 1.02)	1.01 (1.00 to 1.02)
Received DOT	--	--			
No	--	--	1.0 (reference)	1.0 (reference)	1.0 (reference)
Yes	--	--	1.39 (0.59 to 3.28)	1.94 (0.97 to 3.88)	0.62 (0.42 to 0.92)
TB Infection Regimen	--		--	--	--
Mono-Rifampin	--	1.0 (reference)	--	--	--
Isoniazid Containing	--	1.36 (1.05 to 1.77)	--	--	--
MDR-TB Resistance Pattern	--	--	--	--	
MDR-TB	--	--	--	--	1.0 (reference)
Fluoroquinolone and/or SLI Resistance	--	--	--	--	1.54 (1.11 to 2.13)

*No one with HIV.

Abbreviations: TB, tuberculosis; DS-TB, drug susceptible tuberculosis; INHR-TB, isoniazid-resistant tuberculosis; MDR-TB, multidrug resistant tuberculosis; DOT, directly observed therapy; SLI, second-line injectable

Appendix Table 10. Multivariable Analysis of Characteristics Associated with Increasing or Decreasing Costs Among All Patients (TB Infection Excluded), Reported as Cost Ratios and 95% Confidence Intervals

Characteristic	Univariable Analysis (n=223)	Multivariable Analysis (n=223)
TB Type		
DS-TB	1.0 (reference)	1.0 (reference)
INHR-TB	1.63 (1.24 to 2.16)	1.33 (1.05 to 1.68)
MDR-TB	9.17 (6.77 to 12.42)	3.64 (2.61 to 5.08)
Age		
<40 years	1.0 (reference)	1.0 (reference)
≥40 years	0.72 (0.51 to 1.01)	0.94 (0.76 to 1.16)
Sex		
Female	1.0 (reference)	1.0 (reference)
Male	0.78 (0.56 to 1.09)	0.79 (0.65 to 0.97)
HIV		
HIV-Negative or unknown	1.0 (reference)	--
HIV-Positive	2.64 (0.47 to 14.8)	--
Diabetes		
No Diabetes or unknown	1.0 (reference)	--
Has Diabetes	1.11 (0.7 to 1.75)	--
Adverse Events		
None	1.0 (reference)	1.0 (reference)
At Least One	2.48 (1.82 to 3.4)	1.35 (1.08 to 1.68)
Hospitalization		
None or <2 months	1.0 (reference)	1.0 (reference)
≥2 months	6.71 (4.98 to 9.05)	2.61 (1.96 to 3.47)
Acid Fast Bacilli Smear		
Negative or unknown	1.0 (reference)	--
Positive	1.36 (0.97 to 1.89)	--
Cavities on Chest X-Ray		
None or unknown	1.0 (reference)	1.0 (reference)
Yes	1.29 (0.9 to 1.84)	1.3 (1.03 to 1.63)
TB Location		
Pulmonary Only	1.0 (reference)	1.0 (reference)
Extrapulmonary Involvement	0.77 (0.53 to 1.11)	0.96 (0.75 to 1.22)
Number of Contacts		
Per Additional Contact	1.02 (1.01 to 1.03)	1.02 (1.01 to 1.02)
Received DOT		
No	1.0 (reference)	1.0 (reference)
Yes	3.94 (2.27 to 6.85)	1.91 (1.34 to 2.74)

Abbreviations: TB, tuberculosis; DS-TB, drug susceptible tuberculosis; INHR-TB, isoniazid-resistant tuberculosis; MDR-TB, multidrug resistant tuberculosis; DOT, directly observed therapy

Appendix Table 11. Multivariable Analysis of Characteristics Associated with Increasing or Decreasing Costs, Excluding People Who Did Not Complete Treatment, Reported as Cost Ratios and 95% Confidence Intervals

Characteristic	All Patients (n=272)	TB Infection (n=77)	DS-TB (n=83)	INHR-TB (n=63)	MDR-TB (n=49)
TB Type		--	--	--	--
DS-TB	1.0 (reference)	--	--	--	--
TB Infection	0.11 (0.08 to 0.13)	--	--	--	--
INHR-TB	1.8 (1.4 to 2.31)	--	--	--	--
MDR-TB	8.06 (6.02 to 10.81)	--	--	--	--
Age					
<40 years	1.0 (reference)	1.0 (reference)	1.0 (reference)	1.0 (reference)	1.0 (reference)
≥40 years	0.95 (0.78 to 1.15)	1.20 (1.04 to 1.39)	0.82 (0.55 to 1.2)	1.11 (0.82 to 1.52)	0.93 (0.7 to 1.24)
Sex					
Female	1.0 (reference)	1.0 (reference)	1.0 (reference)	1.0 (reference)	1.0 (reference)
Male	0.97 (0.81 to 1.16)	1.0 (0.87 to 1.16)	0.83 (0.59 to 1.17)	1.05 (0.77 to 1.42)	1.0 (0.74 to 1.36)
HIV					
HIV-Negative or unknown	1.0 (reference)	--*	1.0 (reference)	--*	--*
HIV-Positive	6.71 (1.5 to 29.95)	--*	12.76 (2.99 to 54.41)	--*	--*
Diabetes					
No Diabetes or unknown	1.0 (reference)	1.0 (reference)	1.0 (reference)	1.0 (reference)	1.0 (reference)
Has Diabetes	--†	--†	--†	1.31 (0.83 to 2.07)	--†
Adverse Events					
None	1.0 (reference)	1.0 (reference)	1.0 (reference)	1.0 (reference)	1.0 (reference)
At Least One	1.57 (1.26 to 1.97)	--†	1.62 (1.14 to 2.3)	1.23 (0.89 to 1.69)	--†
Hospitalization	--	--			
None or <2 months	--	--	1.0 (reference)	1.0 (reference)	1.0 (reference)
≥2 months	--	--	3.64 (1.84 to 7.18)	3.4 (2.29 to 5.06)	1.43 (1.01 to 2.03)
Acid Fast Bacilli Smear	--	--			
Negative or unknown	--	--	1.0 (reference)	1.0 (reference)	1.0 (reference)
Positive	--	--	1.52 (1.01 to 2.32)	1.34 (0.96 to 1.85)	1.01 (0.73 to 1.41)
Cavities on Chest X-Ray	--	--			
None or unknown	--	--	1.0 (reference)	1.0 (reference)	1.0 (reference)
Yes	--	--	1.25 (0.84 to 1.84)	0.85 (0.6 to 1.19)	1.29 (0.9 to 1.84)
TB Location	--	--			
Pulmonary Only	--	--	1.0 (reference)	1.0 (reference)	1.0 (reference)
Extrapulmonary Involvement	--	--	0.7 (0.47 to 1.04)	--†	--†
Number of Contacts	--	--			
Per Additional Contact	--	--	1.05 (1.02 to 1.08)	1.02 (1.01 to 1.02)	1.01 (1.01 to 1.02)
Received DOT	--	--			
No	--	--	1.0 (reference)	1.0 (reference)	1.0 (reference)
Yes	--	--	--†	1.81 (1.1 to 2.98)	0.88 (0.51 to 1.5)
TB Infection Regimen	--		--	--	--
Mono-Rifampin	--	1.0 (reference)	--	--	--
Isoniazid Containing	--	1.26 (0.98 to 1.61)	--	--	--
MDR-TB Resistance Pattern	--	--	--	--	
MDR-TB	--	--	--	--	1.0 (reference)
Fluoroquinolone and/or SLI Resistance	--	--	--	--	1.37 (0.93 to 2.01)

*No one with HIV.
†Not retained in multivariable model
Abbreviations: TB, tuberculosis; DS-TB, drug susceptible tuberculosis; INHR-TB, isoniazid-resistant tuberculosis; MDR-TB, multidrug resistant tuberculosis; DOT, directly observed therapy; SLI, second-line injectable

Appendix Table 12. TB Infection Patient Characteristics and Costs Stratified by TB Treatment Centre

Characteristic	British Columbia Centre for Disease Control	West Park Healthcare Centre (Ontario)	Montreal Chest Institute (Quebec)
Number of Patients	30	30	30
Regimen			
Isoniazid Only	25 (83%)	23 (77%)	1 (3%)
Rifampin Only	4 (13%)	2 (7%)	29 (97%)
Isoniazid and Rifampin	0 (0%)	2 (7%)	0 (0%)
Started Isoniazid but Switched to Rifampin	1 (3%)	3 (10%)	0 (0%)
Demographic Characteristics			
Median (IQR) Age, years	53 (IQR: 36 to 65)	32 (IQR: 28 to 41)	32 (IQR: 24 to 42)
Male Sex	16 (53%)	6 (20%)	13 (43%)
Female Sex	14 (47%)	24 (80%)	17 (57%)
Born Outside Canada	25 (83%)	26 (87%)	28 (93%)
Clinical Characteristics			
HIV-Positive	0 (0%)	0 (0%)	0 (0%)
HIV-Negative	4 (13%)	29 (97%)	0 (0%)
Unknown HIV Status	26 (87%)	1 (3%)	30 (100%)
Has Diabetes	10 (33%)	2 (7%)	0 (0%)
No Diabetes	17 (57%)	27 (90%)	30 (100%)
Unknown Diabetes	3 (10%)	1 (3%)	0 (0%)
Current Smoker	0 (0%)	5 (17%)	2 (7%)
Ex-Smoker	2 (7%)	4 (13%)	1 (3%)
Never Smoker	19 (63%)	21 (70%)	26 (87%)
Smoking Unknown	9 (30%)	0 (0%)	1 (3%)
Currently Drinks ≥3 drinks per day	0 (0%)	1 (3%)	0 (0%)
Currently Drinks <3 drinks per day	23 (77%)	27 (90%)	29 (97%)
Drinking Habits Unknown	7 (23%)	2 (7%)	1 (3%)
Uses Illicit Drugs	0 (0%)	0 (0%)	0 (0%)
Does not Use Illicit Drugs	21 (70%)	29 (97%)	28 (93%)
Illicit Drug Use Unknown	9 (30%)	1 (3%)	2 (7%)
Treatment Information			
Median (IQR) Treatment Duration, months	9 (IQR: 8.6 to 9.2)	8.7 (IQR: 4 to 9)	4 (IQR: 4 to 4.2)
Cure or Treatment Complete	30 (100%)	21 (70%)	26 (87%)
Incomplete Treatment due to Adverse Event	0 (0%)	4 (13%)	0 (0%)
Incomplete Treatment due to Failure	0 (0%)	0 (0%)	0 (0%)
Lost to Follow-up	0 (0%)	5 (17%)	4 (13%)
Died during Treatment	0 (0%)	0 (0%)	0 (0%)
Cost Information			
Median (IQR) Total Costs	\$798 (IQR: \$682 to \$984)	\$1189 (IQR: \$1033 to \$1505)	\$587 (IQR: \$520 to \$667)
Median (IQR) Cost of Diagnosis	\$252 (IQR: \$217 to \$414)	\$337 (IQR: \$337 to \$399)	\$194 (IQR: \$194 to \$217)
Median (IQR) Cost of Treatment	\$530 (IQR: \$394 to \$691)	\$778 (IQR: \$558 to \$1085)	\$391 (IQR: \$311 to \$473)
Median (IQR) Cost of Post-Treatment Monitoring	\$0 (IQR: \$0 to \$0)	\$18 (IQR: \$0 to \$93)	\$0 (IQR: \$0 to \$0)
Median (IQR) Cost of Hospitalization	\$0 (IQR: \$0 to \$0)	\$0 (IQR: \$0 to \$0)	\$0 (IQR: \$0 to \$0)

Abbreviations: IQR, interquartile range

Appendix Table 13. TB Infection Patient Characteristics and Costs Stratified by Regimen Received

Characteristic	Isoniazid Only	Rifampin Only	Other Isoniazid-Containing Regimen
Number of Patients	49	35	6*
TB Treatment Centre			
Montreal Chest Institute (Quebec)	1 (2%)	29 (83%)	0 (0%)
Ontario	23 (47%)	2 (6%)	5 (83%)
British Columbia Centre for Disease Control	25 (51%)	4 (11%)	1 (17%)
Demographic Characteristics			
Median (IQR) Age, years	42 (IQR: 31 to 59)	32 (IQR: 26 to 42)	36 (IQR: 34 to 39)
Male Sex	18 (37%)	15 (43%)	2 (33%)
Female Sex	31 (63%)	20 (57%)	4 (67%)
Born Outside Canada	41 (84%)	33 (94%)	5 (83%)
Clinical Characteristics			
HIV-Positive	0 (0%)	0 (0%)	0 (0%)
HIV-Negative	27 (55%)	1 (3%)	5 (83%)
Unknown HIV Status	22 (45%)	34 (97%)	1 (17%)
Has Diabetes	9 (18%)	3 (9%)	0 (0%)
No Diabetes	36 (73%)	32 (91%)	6 (100%)
Unknown Diabetes	4 (8%)	0 (0%)	0 (0%)
Current Smoker	5 (10%)	2 (6%)	0 (0%)
Ex-Smoker	5 (10%)	2 (6%)	0 (0%)
Never Smoker	31 (63%)	29 (83%)	6 (100%)
Smoking Unknown	8 (16%)	2 (6%)	0 (0%)
Currently Drinks ≥3 drinks per day	1 (2%)	0 (0%)	0 (0%)
Currently Drinks <3 drinks per day	41 (84%)	33 (94%)	5 (83%)
Drinking Habits Unknown	7 (14%)	2 (6%)	1 (17%)
Uses Illicit Drugs	0 (0%)	0 (0%)	0 (0%)
Does not Use Illicit Drugs	41 (84%)	32 (91%)	5 (83%)
Illicit Drug Use Unknown	8 (16%)	3 (9%)	1 (17%)
Treatment Information			
Median (IQR) Treatment Duration, months	9 (IQR: 8.7 to 9.2)	4 (IQR: 3.9 to 4.2)	4.9 (IQR: 4.1 to 5.9)
Cure or Treatment Complete	42 (86%)	30 (86%)	5 (83%)
Incomplete Treatment due to Adverse Event	3 (6%)	0 (0%)	1 (17%)
Incomplete Treatment due to Failure	0 (0%)	0 (0%)	0 (0%)
Lost to Follow-up	4 (8%)	5 (14%)	0 (0%)
Died during Treatment	0 (0%)	0 (0%)	0 (0%)
Cost Information			
Median (IQR) Total Costs	$985 (IQR: $726 to $1378)	$587 (IQR: $524 to $699)	$1165 (IQR: $1065 to $1242)
Median (IQR) Cost of Diagnosis	$337 (IQR: $217 to $414)	$194 (IQR: $194 to $221)	$379 (IQR: $329 to $424)
Median (IQR) Cost of Treatment	$600 (IQR: $427 to $900)	$392 (IQR: $304 to $478)	$743 (IQR: $709 to $782)
Median (IQR) Cost of Post-Treatment Monitoring	$0 (IQR: $0 to $18)	$0 (IQR: $0 to $0)	$9 (IQR: $0 to $18)
Median (IQR) Cost of Hospitalization	$0 (IQR: $0 to $0)	$0 (IQR: $0 to $0)	$0 (IQR: $0 to $0)

Abbreviations: IQR, interquartile range

*Two patients initiating isoniazid and rifampin regimen, four patients initiating isoniazid and experiencing an adverse event and receiving rifampin.

Appendix Table 14. Drug-Susceptible TB Disease Patient Characteristics and Costs Stratified by TB Treatment Centre

Characteristic	British Columbia Centre for Disease Control	West Park Healthcare Centre (Ontario)	Montreal Chest Institute (Quebec)
Number of Patients	30	30	30
Demographic Characteristics			
Median (IQR) Age, years	56 (IQR: 39 to 78)	50 (IQR: 38 to 76)	30 (IQR: 27 to 37)
Male Sex	12 (40%)	19 (63%)	9 (30%)
Female Sex	18 (60%)	11 (37%)	21 (70%)
Born Outside Canada	26 (87%)	27 (90%)	27 (90%)
Clinical Characteristics			
HIV-Positive	0 (0%)	0 (0%)	1 (3%)
HIV-Negative	24 (80%)	23 (77%)	22 (73%)
Unknown HIV Status	6 (20%)	7 (23%)	7 (23%)
Has Diabetes	3 (10%)	8 (27%)	2 (7%)
No Diabetes	25 (83%)	22 (73%)	28 (93%)
Unknown Diabetes	2 (7%)	0 (0%)	0 (0%)
Current Smoker	5 (17%)	7 (23%)	2 (7%)
Ex-Smoker	1 (3%)	4 (13%)	0 (0%)
Never Smoker	21 (70%)	19 (63%)	28 (93%)
Smoking Unknown	3 (10%)	0 (0%)	0 (0%)
Currently Drinks ≥3 drinks per day	1 (3%)	0 (0%)	0 (0%)
Currently Drinks <3 drinks per day	24 (80%)	28 (93%)	30 (100%)
Drinking Habits Unknown	5 (17%)	2 (7%)	0 (0%)
Uses Illicit Drugs	0 (0%)	1 (3%)	0 (0%)
Does not Use Illicit Drugs	26 (87%)	27 (90%)	30 (100%)
Illicit Drug Use Unknown	4 (13%)	2 (7%)	0 (0%)
Disease Characteristics			
Acid Fast Bacilli Smear Positive	19 (63%)	21 (70%)	7 (23%)
Acid Fast Bacilli Smear Negative	11 (37%)	9 (30%)	23 (77%)
Acid Fast Bacilli Smear Unknown	0 (0%)	0 (0%)	0 (0%)
Cavities on Chest X-Ray	11 (37%)	12 (40%)	7 (23%)
No Cavities on Chest X-Ray	18 (60%)	18 (60%)	23 (77%)
Unknown Cavities	1 (3%)	0 (0%)	0 (0%)
Exclusively Pulmonary Disease	22 (73%)	21 (70%)	25 (83%)
Disease with Extrapulmonary Involvement	8 (27%)	9 (30%)	5 (17%)
Treatment Information			
Hospitalized	15 (50%)	23 (77%)	8 (27%)
Median (IQR) Duration of Hospitalization, days	37 (IQR: 26 to 61)	13 (IQR: 8 to 27)	24 (IQR: 14 to 28)
Median (IQR) Drugs Stopped for Adverse Event	0 (IQR: 0 to 4)	1 (IQR: 0 to 1)	0 (IQR: 0 to 1)
Received a TB Drug Other than Isoniazid, Rifampin, Ethambutol, and Pyrazinamide	10 (33%)	4 (13%)	7 (23%)
Median (IQR) Treatment Duration, months	8.9 (IQR: 6.2 to 9.7)	9.1 (IQR: 7.6 to 10)	6.4 (IQR: 6.1 to 9)
Cure or Treatment Complete	26 (87%)	27 (90%)	30 (100%)
Incomplete Treatment due to Adverse Event	0 (0%)	0 (0%)	0 (0%)
Incomplete Treatment due to Failure	0 (0%)	0 (0%)	0 (0%)
Lost to Follow-up	2 (7%)	3 (10%)	0 (0%)
Died during Treatment	2 (7%)	0 (0%)	0 (0%)
Cost Information			
Median (IQR) Total Costs	$15,201 (IQR: $6975 to $33,983)	$13,328 (IQR: $7921 to $19,080)	$4987 (IQR: $3572 to $16,196)
Median (IQR) Cost of Diagnosis	$962 (IQR: $671 to $1165)	$653 (IQR: $506 to $742)	$615 (IQR: $454 to $987)
Median (IQR) Cost of Treatment	$2642 (IQR: $1996 to $4138)	$1951 (IQR: $1444 to $2158)	$2071 (IQR: $1708 to $3319)
Median (IQR) Cost of Post-Treatment Monitoring	$115 (IQR: $0 to $230)	$141 (IQR: $67 to $252)	$107 (IQR: $28 to $435)
Median (IQR) Cost of Hospitalization	$975 (IQR: $0 to $23,888)	$5850 (IQR: $3250 to $11,212)	$0 (IQR: $0 to $3423)
Median (IQR) Cost for Public Health Interventions	$3174 (IQR: $1731 to $5626)	$5213 (IQR: $705 to $5353)	$1154 (IQR: $238 to $3462)

Abbreviations: IQR, interquartile range

Appendix Table 15. Isoniazid-Resistant TB Disease Patient Characteristics and Costs Stratified by TB Treatment Centre

Characteristic	British Columbia Centre for Disease Control	West Park Healthcare Centre (Ontario)	Montreal Chest Institute (Quebec)
Number of Patients	30	27	14
Demographic Characteristics			
Median (IQR) Age, years	52 (IQR: 34 to 69)	41 (IQR: 30 to 57)	39 (IQR: 31 to 54)
Male Sex	14 (47%)	12 (44%)	7 (50%)
Female Sex	16 (53%)	15 (56%)	7 (50%)
Born Outside Canada	27 (90%)	25 (93%)	10 (71%)
Clinical Characteristics			
HIV-Positive	0 (0%)	0 (0%)	0 (0%)
HIV-Negative	0 (0%)	0 (0%)	12 (86%)
Unknown HIV Status	30 (100%)	27 (100%)	2 (14%)
Has Diabetes	6 (20%)	3 (11%)	1 (7%)
No Diabetes	24 (80%)	24 (89%)	12 (86%)
Unknown Diabetes	0 (0%)	0 (0%)	1 (7%)
Current Smoker	4 (13%)	5 (19%)	2 (14%)
Ex-Smoker	2 (7%)	5 (19%)	2 (14%)
Never Smoker	22 (73%)	15 (56%)	9 (64%)
Smoking Unknown	2 (7%)	2 (7%)	1 (7%)
Currently Drinks ≥3 drinks per day	3 (10%)	3 (11%)	0 (0%)
Currently Drinks <3 drinks per day	23 (77%)	22 (81%)	12 (86%)
Drinking Habits Unknown	4 (13%)	2 (7%)	2 (14%)
Uses Illicit Drugs	0 (0%)	0 (0%)	0 (0%)
Does not Use Illicit Drugs	25 (83%)	26 (96%)	13 (93%)
Illicit Drug Use Unknown	5 (17%)	1 (4%)	1 (7%)
Disease Characteristics			
Acid Fast Bacilli Smear Positive	9 (30%)	9 (33%)	3 (21%)
Acid Fast Bacilli Smear Negative	21 (70%)	18 (67%)	6 (43%)
Acid Fast Bacilli Smear Unknown	0 (0%)	0 (0%)	5 (36%)
Cavities on Chest X-Ray	16 (53%)	12 (44%)	7 (50%)
No Cavities on Chest X-Ray	14 (47%)	15 (56%)	7 (50%)
Unknown Cavities	0 (0%)	0 (0%)	0 (0%)
Exclusively Pulmonary Disease	18 (60%)	22 (81%)	11 (79%)
Disease with Extrapulmonary Involvement	12 (40%)	5 (19%)	3 (21%)
Treatment Information			
Hospitalized	17 (57%)	25 (93%)	5 (36%)
Median (IQR) Duration of Hospitalization, days	19 (IQR: 13 to 51)	54 (IQR: 20 to 76)	7 (IQR: 5 to 20)
Median (IQR) Drugs Stopped for Adverse Event	0 (IQR: 0 to 1)	0 (IQR: 0 to 1)	0 (IQR: 0 to 0)
Received a Fluoroquinolone	21 (70%)	27 (100%)	6 (43%)
Received a Second-Line Injectable	0 (0%)	8 (30%)	0 (0%)
Median (IQR) Treatment Duration, months	11.5 (IQR: 9.3 to 12.4)	17.6 (IQR: 12.3 to 18.8)	8 (IQR: 6.1 to 9.4)
Cure or Treatment Complete	24 (80%)	25 (93%)	14 (100%)
Incomplete Treatment due to Adverse Event	0 (0%)	0 (0%)	0 (0%)
Incomplete Treatment due to Failure	2 (7%)	0 (0%)	0 (0%)
Lost to Follow-up	2 (7%)	2 (7%)	0 (0%)
Died during Treatment	2 (7%)	0 (0%)	0 (0%)
Cost Information			
Median (IQR) Total Costs	$12,506 (IQR: $5652 to $26,443)	$34,400 (IQR: $22,391 to $63,222)	$6504 (IQR: $5156 to $9761)
Median (IQR) Cost of Diagnosis	$858 (IQR: $674 to $1144)	$785 (IQR: $637 to $1002)	$806 (IQR: $651 to $883)
Median (IQR) Cost of Treatment	$2407 (IQR: $1871 to $3226)	$3835 (IQR: $2464 to $6943)	$2831 (IQR: $2391 to $3582)
Median (IQR) Cost of Post-Treatment Monitoring	$132 (IQR: $0 to $163)	$127 (IQR: $60 to $195)	$122 (IQR: $107 to $563)
Median (IQR) Cost of Hospitalization	$3250 (IQR: $0 to $13,812)	$23,400 (IQR: $12,025 to $43,875)	$0 (IQR: $0 to $2969)
Median (IQR) Cost for Public Health Interventions	$2020 (IQR: $578 to $5049)	$6115 (IQR: $5493 to $6218)	$1442 (IQR: $24 to $2885)

Abbreviations: IQR, interquartile range

Appendix Table 16. Multidrug-Resistant TB Disease Patient Characteristics and Costs Stratified by TB Treatment Centre

Characteristic	British Columbia Centre for Disease Control	West Park Healthcare Centre (Ontario)	Montreal Chest Institute (Quebec)
Number of Patients	11	45	6
Demographic Characteristics			
Median (IQR) Age, years	41 (IQR: 31 to 46)	31 (IQR: 27 to 46)	39 (IQR: 34 to 49)
Male Sex	4 (36%)	24 (53%)	0 (0%)
Female Sex	7 (64%)	21 (47%)	6 (100%)
Born Outside Canada	10 (91%)	41 (91%)	6 (100%)
Clinical Characteristics			
HIV-Positive	0 (0%)	1 (2%)	0 (0%)
HIV-Negative	10 (91%)	44 (98%)	5 (83%)
Unknown HIV Status	1 (9%)	0 (0%)	1 (17%)
Has Diabetes	1 (9%)	8 (18%)	1 (17%)
No Diabetes	10 (91%)	37 (82%)	5 (83%)
Unknown Diabetes	0 (0%)	0 (0%)	0 (0%)
Current Smoker	5 (45%)	10 (22%)	0 (0%)
Ex-Smoker	0 (0%)	9 (20%)	0 (0%)
Never Smoker	6 (55%)	26 (58%)	6 (100%)
Smoking Unknown	0 (0%)	0 (0%)	0 (0%)
Currently Drinks ≥3 drinks per day	3 (27%)	17 (38%)	0 (0%)
Currently Drinks <3 drinks per day	8 (73%)	27 (60%)	6 (100%)
Drinking Habits Unknown	0 (0%)	1 (2%)	0 (0%)
Uses Illicit Drugs	0 (0%)	1 (2%)	0 (0%)
Does not Use Illicit Drugs	11 (100%)	44 (98%)	6 (100%)
Illicit Drug Use Unknown	0 (0%)	0 (0%)	0 (0%)
Disease Characteristics			
Acid Fast Bacilli Smear Positive	5 (45%)	9 (20%)	1 (17%)
Acid Fast Bacilli Smear Negative	6 (55%)	36 (80%)	5 (83%)
Acid Fast Bacilli Smear Unknown	0 (0%)	0 (0%)	0 (0%)
Cavities on Chest X-Ray	7 (64%)	14 (31%)	1 (17%)
No Cavities on Chest X-Ray	4 (36%)	31 (69%)	5 (83%)
Unknown Cavities	0 (0%)	0 (0%)	0 (0%)
Exclusively Pulmonary Disease	9 (82%)	35 (78%)	3 (50%)
Disease with Extrapulmonary Involvement	2 (18%)	10 (22%)	3 (50%)
Resistant to Fluoroquinolone or Second-Line Injectable	1 (9%)	10 (22%)	1 (17%)
Treatment Information			
Hospitalized	10 (91%)	45 (100%)	5 (83%)
Median (IQR) Duration of Hospitalization, days	72 (IQR: 55 to 136)	106 (IQR: 85 to 166)	49 (IQR: 34 to 94)
Median (IQR) Drugs Stopped for Adverse Event	2 (IQR: 2 to 2)	2 (IQR: 1 to 3)	1 (IQR: 1 to 2)
Received Bedaquiline	1 (9%)	2 (4%)	0 (0%)
Received Linezolid	5 (45%)	24 (53%)	5 (83%)
Received Delamanid	2 (18%)	0 (0%)	2 (33%)
Median (IQR) Treatment Duration, months	20.8 (IQR: 20.1 to 21.6)	22.7 (IQR: 20.1 to 25.8)	19.2 (IQR: 18.3 to 20)
Cure or Treatment Complete	9 (82%)	35 (78%)	5 (83%)
Incomplete Treatment due to AE	1 (9%)	0 (0%)	0 (0%)
Incomplete Treatment due to Failure	0 (0%)	0 (0%)	0 (0%)
Lost to Follow-up	1 (9%)	8 (18%)	1 (17%)
Died during Treatment	0 (0%)	2 (4%)	0 (0%)
Cost Information			
Median (IQR) Total Costs	$153,086 (IQR: $144,553 to $232,607)	$107,955 (IQR: $78,951 to $148,983)	$116,751 (IQR: $113,925 to $136,444)
Median (IQR) Cost of Diagnosis	$892 (IQR: $740 to $1218)	$1102 (IQR: $1007 to $1343)	$742 (IQR: $677 to $795)
Median (IQR) Cost of Treatment	$134,021 (IQR: $83,946 to $162,725)	$41,513 (IQR: $29,301 to $100,296)	$79,240 (IQR: $32,554 to $106,371)
Median (IQR) Cost of Post-Treatment Monitoring	$300 (IQR: $164 to $519)	$158 (IQR: $0 to $286)	$398 (IQR: $340 to $472)
Median (IQR) Cost of Hospitalization	$40,952 (IQR: $26,977 to $51,778)	$42,082 (IQR: $37,270 to $56,318)	$30,302 (IQR: $17,247 to $61,265)
Median (IQR) Cost for Public Health Interventions	$4616 (IQR: $1485 to $7338)	$6418 (IQR: $5096 to $6773)	$7058 (IQR: $5842 to $11,431)

Abbreviations: IQR, interquartile range; AE, adverse event

Appendix Table 17. Multidrug-Resistant TB Disease Patient Characteristics and Costs Stratified by Resistance Pattern

Characteristic	Susceptible to both Fluoroquinolones and Second-Line Injectables	Resistant to a Fluoroquinolone and/or Second-Line Injectable
Number of Patients	50	12
Demographic Characteristics		
Median (IQR) Age, years	34 (IQR: 27 to 48)	31 (IQR: 28 to 43)
Male Sex	27 (54%)	1 (8%)
Female Sex	23 (46%)	11 (92%)
Born Outside Canada	46 (92%)	11 (92%)
Clinical Characteristics		
HIV-Positive	1 (2%)	0 (0%)
HIV-Negative	47 (94%)	12 (100%)
Unknown HIV Status	2 (4%)	0 (0%)
Has Diabetes	10 (20%)	0 (0%)
No Diabetes	40 (80%)	12 (100%)
Unknown Diabetes	0 (0%)	0 (0%)
Current Smoker	11 (22%)	4 (33%)
Ex-Smoker	7 (14%)	2 (17%)
Never Smoker	32 (64%)	6 (50%)
Smoking Unknown	0 (0%)	0 (0%)
Currently Drinks ≥3 drinks per day	14 (28%)	6 (50%)
Currently Drinks <3 drinks per day	35 (70%)	6 (50%)
Drinking Habits Unknown	1 (2%)	0 (0%)
Uses Illicit Drugs	1 (2%)	0 (0%)
Does not Use Illicit Drugs	49 (98%)	12 (100%)
Illicit Drug Use Unknown	0 (0%)	0 (0%)
Disease Characteristics		
Acid Fast Bacilli Smear Positive	12 (24%)	3 (25%)
Acid Fast Bacilli Smear Negative	38 (76%)	9 (75%)
Acid Fast Bacilli Smear Unknown	0 (0%)	0 (0%)
Cavities on Chest X-Ray	17 (34%)	5 (42%)
No Cavities on Chest X-Ray	33 (66%)	7 (58%)
Unknown Cavities	0 (0%)	0 (0%)
Exclusively Pulmonary Disease	38 (76%)	9 (75%)
Disease with Extrapulmonary Involvement	12 (24%)	3 (25%)
Treatment Information		
Hospitalized	48 (96%)	12 (100%)
Median (IQR) Duration of Hospitalization, days	95 (IQR: 62 to 145)	152 (IQR: 96 to 170)
Median (IQR) Drugs Stopped for Adverse Event	2 (IQR: 1 to 2)	2 (IQR: 1 to 3)
Received Bedaquiline	1 (2%)	2 (17%)
Received Linezolid	25 (50%)	9 (75%)
Received Delamanid	3 (6%)	1 (8%)
Median (IQR) Treatment Duration, months	20.9 (IQR: 20 to 24.2)	24.5 (IQR: 19.8 to 27)
Cure or Treatment Complete	39 (78%)	10 (83%)
Incomplete Treatment due to Adverse Event	1 (2%)	0 (0%)
Incomplete Treatment due to Failure	0 (0%)	0 (0%)
Lost to Follow-up	8 (16%)	2 (17%)
Died during Treatment	2 (4%)	0 (0%)
Cost Information		
Median (IQR) Total Costs	$113,952 (IQR: $77,813 to $150,573)	$150,150 (IQR: $101,802 to $205,764)
Median (IQR) Cost of Diagnosis	$1083 (IQR: $910 to $1313)	$1108 (IQR: $989 to $1386)
Median (IQR) Cost of Treatment	$61,414 (IQR: $25,411 to $108,289)	$80,710 (IQR: $44,435 to $128,231)
Median (IQR) Cost of Post-Treatment Monitoring	$189 (IQR: $0 to $341)	$219 (IQR: $118 to $338)
Median (IQR) Cost of Hospitalization	$40,815 (IQR: $34,322 to $52,995)	$61,195 (IQR: $39,576 to $104,532)
Median (IQR) Cost for Public Health Interventions	$6383 (IQR: $4657 to $6767)	$6696 (IQR: $5176 to $6880)

Abbreviations: IQR, interquartile range
